# Dual regulatory switch through interactions of Tcf7l2/Tcf4 with stage-specific partners propels oligodendroglial maturation

**DOI:** 10.1038/ncomms10883

**Published:** 2016-03-09

**Authors:** Chuntao Zhao, Yaqi Deng, Lei Liu, Kun Yu, Liguo Zhang, Haibo Wang, Xuelian He, Jincheng Wang, Changqing Lu, Laiman N Wu, Qinjie Weng, Meng Mao, Jianrong Li, Johan H van Es, Mei Xin, Lee Parry, Steven A Goldman, Hans Clevers, Q. Richard Lu

**Affiliations:** 1 Department of Pediatrics, State Key Laboratory of Biotherapy, West China Second Hospital, Sichuan University, Collaborative Innovation Center for Biotherapy, Chengdu 610041, China; 2 Division of Experimental Hematology and Cancer Biology, Department of Pediatrics, Brain Tumor Center, Cincinnati Children's Hospital Medical Center, Cincinnati, Ohio 45229, USA; 3 Key Laboratory of Obstetrics, and Gynecologic and Pediatric Diseases and Birth Defects of Ministry of Education, West China Second Hospital, Sichuan University, Collaborative Innovation Center for Biotherapy, Chengdu 610041, China; 4 Institute of Pharmacology and Toxicology, College of Pharmaceutical Sciences, Zhejiang University, Hangzhou 310058, China; 5 Department of Veterinary Integrative Biosciences, Texas A&M University, College Station, Texas 77843, USA; 6 Hubrecht Institute, Uppsalalaan 8, Utrecht 3584CT, The Netherlands; 7 European Cancer Stem Cell Research Institute, Cardiff University, Cardiff CF244HQ, UK; 8 Center for Translational Neuromedicine, University of Rochester Medical Center, 601 Elmwood Avenue Rochester, New York 14642, USA; 9 Key Laboratory of Birth Defects, Children's Hospital of Fudan University, Shanghai 201102, China

## Abstract

Constitutive activation of Wnt/β-catenin inhibits oligodendrocyte myelination. Tcf7l2/Tcf4, a β-catenin transcriptional partner, is required for oligodendrocyte differentiation. How Tcf7l2 modifies β-catenin signalling and controls myelination remains elusive. Here we define a stage-specific Tcf7l2-regulated transcriptional circuitry in initiating and sustaining oligodendrocyte differentiation. Multistage genome occupancy analyses reveal that Tcf7l2 serially cooperates with distinct co-regulators to control oligodendrocyte lineage progression. At the differentiation onset, Tcf7l2 interacts with a transcriptional co-repressor Kaiso/Zbtb33 to block β-catenin signalling. During oligodendrocyte maturation, Tcf7l2 recruits and cooperates with Sox10 to promote myelination. In that context, Tcf7l2 directly activates cholesterol biosynthesis genes and cholesterol supplementation partially rescues oligodendrocyte differentiation defects in *Tcf712* mutants. Together, we identify stage-specific co-regulators Kaiso and Sox10 that sequentially interact with Tcf7l2 to coordinate the switch at the transitions of differentiation initiation and maturation during oligodendrocyte development, and point to a previously unrecognized role of Tcf7l2 in control of cholesterol biosynthesis for CNS myelinogenesis.

## Abstract (web summary)

Wnt/β-catenin signaling regulates oligodendrocyte (OL) development. Here the authors show that Tcf7l2, a β-catenin transcriptional partner,sequentially interacts with stage-specific partners to coordinate the transitions of differentiation initiation and maturation during OL development.

## Graphical abstract

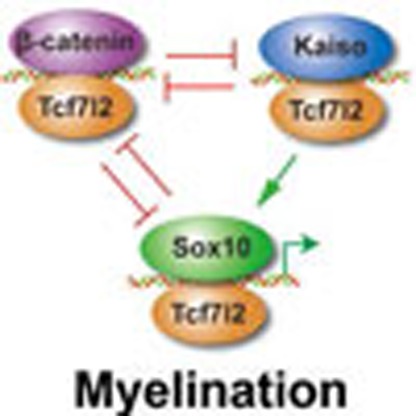

Oligodendrocyte (OL) myelination permits saltatory propagation of nerve signals and is critical for cognitive and motor functions in the vertebrate central nervous system (CNS)^1^,^2^,^3^,^4^,^5^. During myelination, OLs pass through multiple developmental stages, including OL precursor cell (OPC), immature premyelinating OL and mature myelinating OL stages. A series of signalling pathways including Wnt/β-catenin, BMP/Id and Notch/Hes signalling have been shown to negatively regulate OL differentiation^6^,^7^. Hyperactivation of canonical Wnt signalling leads to the inhibition of OL differentiation and myelination through constitutively activated β-catenin^8^,^9^,^10^, Wnt3a ligand treatment^11^,^12^,^13^ or the loss of signalling inhibitors, as observed in Apc^Min^ (ref. 14) or Apc knockout mice^15^. In addition to these signalling pathways that sense the presence of extrinsic factors in the environment, intrinsic factors such as transcription factors including *Olig1*/2, Sox10, Zeb2/Sip1, Yy1, Zfp191 and Myrf/Gm98 positively regulate OL development^6^,^7^. Despite the critical roles of these signalling pathways and transcriptional regulators in OL differentiation, the means by which different signalling pathways and transcriptional regulatory circuitries are integrated to control OL differentiation remains poorly understood.

Activation of Wnt signalling on Wnt ligand binding results in the stabilization and subsequent nuclear translocation of β-catenin^16^. Nuclear β-catenin binds to T-cell factor (TCF)/lymphoid enhancer-binding factors including Tcf7l2 (a.k.a. Tcf4). The TCF complex with nuclear β-catenin activates Wnt target genes^16^ and β-catenin-mediated transcriptional activation is principally through TCFs^17^. Tcf7l2 is a major transducer of β-catenin activity^17^ and is highly expressed by OL lineage cells^8^,^9^. Although hyperactive Wnt signalling inhibits OL differentiation, intriguingly, the loss of the β-catenin effector Tcf7l2 leads to a block of OL differentiation in *Tcf7l2*-null animals^9^,^18^. At present, the mechanisms underlying Tcf7l2 regulation of CNS myelination and remyelination remain elusive. Importantly, direct transcriptional targets of Tcf7l2 have not been identified in OLs, a particular concern, as Tcf7l2 is a known partner of β-catenin, raising the paradox of how Tcf7l2 might exert functions opposing inhibitory functions of β-catenin.

By generating mice lacking the Tcf7l2 DNA-binding transcription-activating domain, we showed that Tcf7l2 transcriptional activity is crucial for OL myelination and remyelination. We further conducted genome-wide chromatin immunoprecipitation sequencing (ChIP-seq) profiling to comprehensively map the Tcf7l2 direct targets at different stages of OL development and find that Tcf7l2 engages the OL genome through its sequential interactions with stage-specific co-regulators, including the non-canonical Wnt signalling repressor Kaiso/Zbtb33 at the early phase of OPC differentiation and a differentiation-promoting factor, Sox10, later in OL differentiation. Our data further suggest that Tcf7l2 interacts with Kaiso to antagonize Wnt signalling activity at the differentiation onset, while coordinating with Sox10 to promote myelin gene expression during OL maturation. Furthermore, we find that Tcf7l2 and Sox10 interaction controls the cholesterol biosynthesis pathway for myelinogenesis. Thus, our studies define stage-dependent functions of Tcf7l2 during OL lineage development mediated through switching binding partners and provide a molecular framework for understanding the context-specific control of CNS myelination.

## Results

### Tcf7l2 transcriptional activity is vital for OL myelination

**Figure 1:**
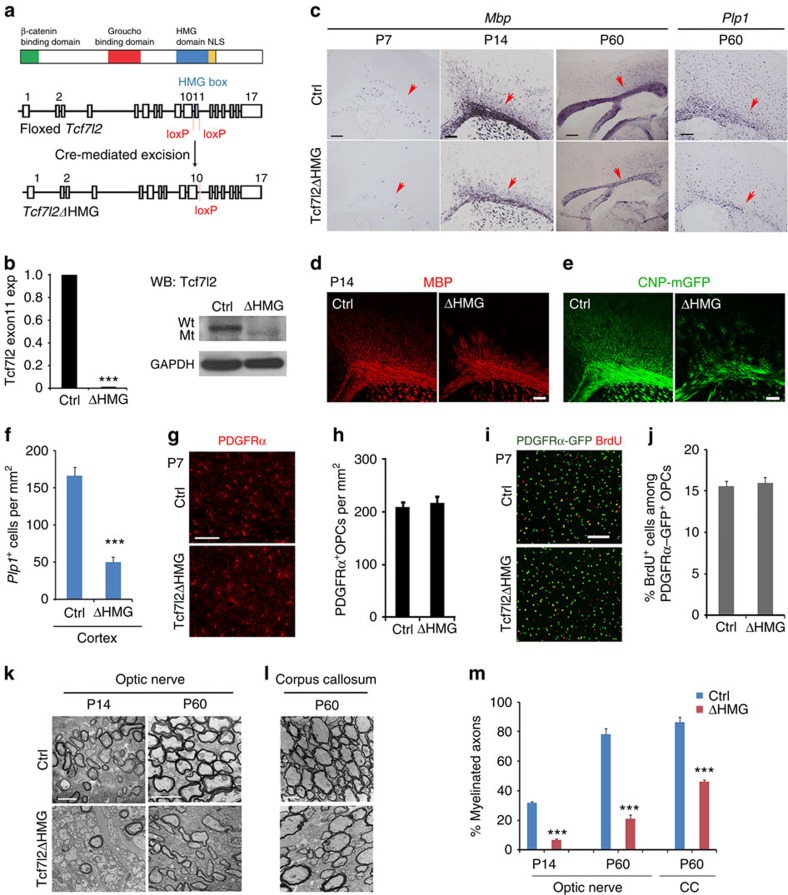
OL differentiation defects in the brain of Tcf7l2ΔHMG mice lacking the DNA-binding HMG domain. (**a**) Schematic diagram shows domains in *Tcf7l2* (upper) and Cre-mediated excision (lower) of the floxed exon 11, which encodes the DNA-binding HMG domain. NLS: Nuclear localization sequence. (**b**) Upper panel, expression of *Tcf7l2* exon 11 in the mRNAs of OPCs isolated from control (Ctrl) and *Tcf7l2*ΔHMG brains at P2 was assayed by qRT–PCR (*n*=3 animals per genotype). Lower panel: western blot analysis of the spinal cords from control and *Tcf7l2*ΔHMG mice at P10 with anti-Tcf7l2 (amino-terminal epitope) and glyceraldehydes 3-phosphate dehydrogenase (GAPDH; loading control). (**c**) Expression of *Mbp* and *Plp1* in cortices from control and *Tcf7l2*ΔHMG mice by *in situ* hybridization. Arrows indicate the cerebral white matter in coronal sections, except P60 *Mbp* in sagittal sections. (**d**) MBP immunostaining in the cortices from control and *Tcf7l2*ΔHMG mice at P14 assayed by immunohistochemistry. (**e**) Expression of membrane-anchored enhanced GFP (EGFP) driven by a CNP promoter in the cortices from control and *Tcf7l2*ΔHMG mice carrying the *CNP*-mGFP transgene at P14. (**f**) Quantification of *Plp1*^+^ OLs (per mm^2^) in the cortex of control and *Tcf7l2*ΔHMG at P60; *n*=3 animals per genotype. (**g**) The cortices of Ctrl and *Tcf7l2*ΔHMG mice at P14 were immunostained with anti-PDGFRα antibody. (**h**) PDGFRα^+^ OPC numbers were quantified per mm^2^; *n*=3 animals per genotype. (**i**) BrdU incorporation in the cortex of control and *Tcf7l2*ΔHMG mice carrying PDGFRα-GFP reporter and pulse labelled with BrdU for 2 h at P14. (**j**) BrdU^+^/PDGFRα^+^ OPC cell numbers were quantified per mm^2^; *n*=3 animals per genotype. (**k**,**l**) Electron microscopy of the optic nerves (OP) and corpus callosum (CC) of control and *Tcf7l2*ΔHMG mice at P14 and P60. (**m**) The percentages of myelinated axons in the OP and CC at P14 and P60; *n*=3 animals per genotype. Data are presented as mean±s.e.m. ***P<0.001; Student's *t*-test. Scale bars, 100 μm (**c**,**d**), 50 μm (**e**,**g**–**j**) and 2 μm (**k**,**l**).

Tcf7l2 consists of several functional domains including the β-catenin-binding domain, Groucho/TLE-binding domain and HMG (high mobility group) DNA-binding domain (Fig. 1a)^19^. To assess the role of Tcf7l2 transcriptional activity during OL development, we generated mutant mice carrying a transcriptionally inactive Tcf7l2, in which the floxed *Tcf7l2* exon 11 encoding the DNA-binding HMG box^20^,^21^ was excised by an OL lineage-expressing *Olig1*-Cre^9^,^22^, to generate a Tcf7l2 in-frame mutant without the HMG domain. This yielded control (Tcf7l2^fl/+^:*Olig1*-Cre^+/−^) and mutant (*Tcf7l2*^fl/fl^: *Olig1*-Cre^+/−^) mice (designated as *Tcf7l2*ΔHMG; Fig. 1a). We confirmed the excision of the exon 11 in complementary DNAs of OPCs isolated from *Tcf7l2*ΔHMG neonates by quantitative reverse transcriptase–PCR (qRT–PCR), using primers spanning the deleted exon 11 (Fig. 1b). Expression of Tcf7l2ΔHMG, but not full-length Tcf7l2 protein, was detected in *Tcf7l2*-mutant spinal cords, although its level is lower when compared with that of control animals by western blot analysis (Fig. 1b).

To investigate OL differentiation in *Tcf7l2* mutants, we first examined myelin gene expression in the brain. In the *Tcf7l2*ΔHMG cortex, we found that in contrast to their robust expression in the control, *Mbp* and *Plp1* (*proteolipid protein 1*) expression was remarkably reduced at postnatal stages (Fig. 1c). The dysmyelinating phenotype persisted to adulthood (Fig. 1c). Myelin basic protein (MBP) expression or green fluorescent protein (GFP) signals from the *CNP*-mGFP transgenic line^23^ was also reduced at P14 (Fig. 1d,e), consistent with the sustained decrease in the number of *Plp1*^+^ OLs at P60 (Fig. 1f).

Impaired terminal OL differentiation in *Tcf7l2* mutants might be due to a shortage in OPCs. We then assessed the formation of cortical OPCs using platelet-derived growth factor receptor-α (PDGFRα) immunolabelling and a *PDGFRα*-GFP reporter^24^. In the *Tcf7l2*ΔHMG cortex at P7, the number of PDGFRα^+^ OPCs was comparable to that in the control (Fig. 1g-j). The rate of OPC proliferation was also unaltered, as shown by 5-bromodeoxyuridine (BrdU) incorporation (Fig. 1i,j). Furthermore, inactivation of *Tcf7l2* in the OL lineage did not affect the generation of neurons, astrocytes or microglia, as immunolabelled by NeuN, glial fibrillary acidic protein and Iba1, respectively (Supplementary Fig. 1). These results indicated that Tcf7l2 is not essential for OPC formation.

In light of OL differentiation deficits noted in the *Tcf7l2* mutants, we used electron microscopy to analyse myelin sheath morphologies in the corpus callosum and in optic nerves. Consistent with the decrease in myelin gene expression in the brain, the optic nerves of mutant mice exhibited a significantly reduced proportion of myelinated axons at early postnatal stages such as P14, this relative hypomyelination persisted into adulthood (Fig. 1k). Similarly, hypomyelination was also observed in the corpus callosum at P60 (Fig. 1l), in which the percentage of myelinated axons remained significantly lower than controls (Fig. 1m). These data suggest that the loss of Tcf7l2 transcriptional activity impairs myelination in both developing and adult brains.

To further verify the stage-specific function of Tcf7l2 in OL differentiation, we inactivated *Tcf7l2* using other OL-lineage expressing Cre drivers, including *Olig2*-Cre, the expression of which begins in early OL progenitors^8^. Similar to *Olig1*-Cre-mediated mutagenesis, ablation of *Tcf7l2* by *Olig2*-Cre resulted in a substantial reduction in the expression of the myelin genes *Mbp* and *Plp1* in the mutant cortex at P14 (Supplementary Fig. 2a). In addition, in mice with *Tcf7l2* ablated by a *CNP*-Cre line^25^, wherein Cre expression commences at early postmitotic OPC stages, there was a significant decrease in the number of CC1^+^ or MAG^+^ myelinating OLs (Supplementary Fig. 2b,c). Together, these observations suggest that Tcf7l2 regulates myelination-associated gene expression and is critical for OPC differentiation.

### Tcf7l2 activity is required for OL remyelination

**Figure 2:**
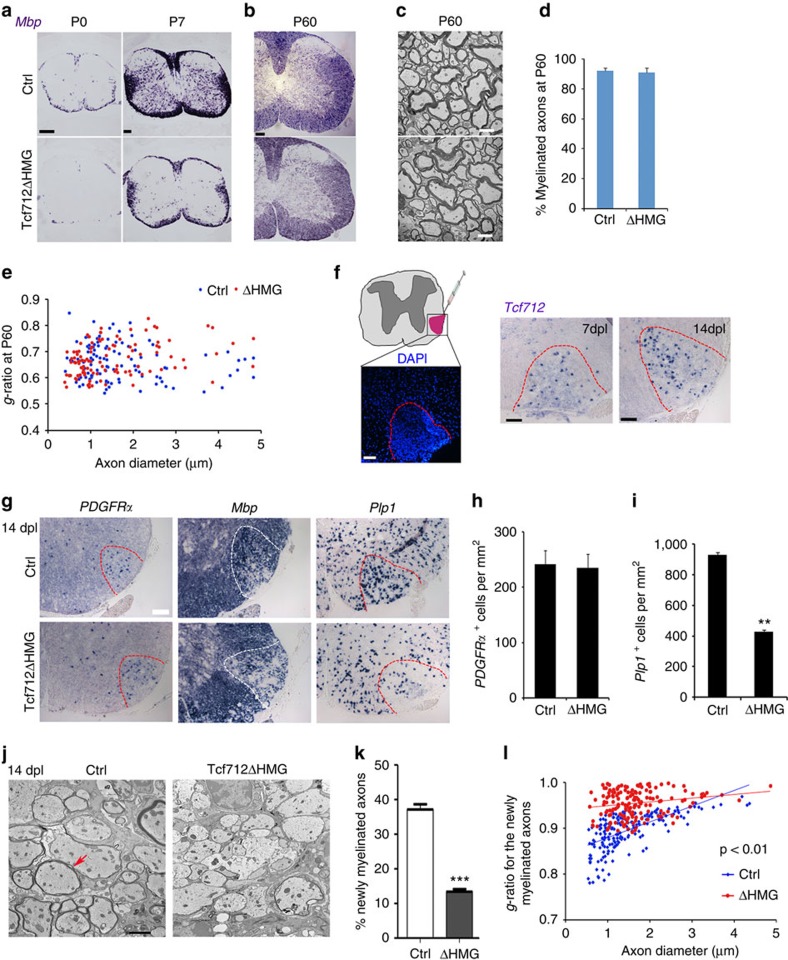
*Tcf7l2* ablation impairs remyelination in LPC-induced demyelinating animal model. (**a**,**b**) Expression of *Mbp* in the spinal cord from control (Ctrl) and *Tcf7l2*ΔHMG mice at indicated neonatal and adult ages by *in situ* hybridization. (**c**) Electron microscopy of the spinal white matter of control and *Tcf7l2*ΔHMG mice at P60. The percentages of myelinated axons (**d**) and *g*-ratio (**e**) in the spinal white matter of control and *Tcf7l2*ΔHMG mice at P60. The data represent the means±s.e.m.; *n*=3 animals per genotype. (**f**) Left: the location of LPC-induced lesion (DAPI counterstaining, dashed lines) in the spinal cord. Right: *in situ* hybridization analysis showed re-expression of *Tcf7l2* in the LPC-induced demyelinating lesions (demarcated with dashed lines) and uninjured regions at 7 and 14 dpl in spinal cords of P60 mice. (**g**) *In situ* hybridization analysis of *PDGFR*α, *Mbp* and *Plp1* in the lesion regions (demarcated with dashed lines) at 14 dpl in spinal cords of P60 Ctrl and *Tcf7l2*ΔHMG mutant mice. Quantification of the numbers of *PDGFR*α^+^ OPC (**h**) and *Plp1*^+^ OL (**i**) at 14 dpl in spinal cords of P60 control and *Tcf7l2*ΔHMG mutant mice; *n*=5 animals for each genotype. (**j**) Representative electron micrographs of spinal cords of 8-week-old control and *Tcf7l2*ΔHMG mice at 14 dpl. Arrow indicates the newly formed thin myelin sheath. (**k**) Quantification of the percentage newly myelinated axons in spinal lesions of 8-week-old control and *Tcf7l2*ΔHMG mice at 14 dpl; *n*=3 animals for each genotype. (**l**) Quantification of *g*-ratio of newly myelinated axons in spinal lesions of 8-week-old control and *Tcf7l2*ΔHMG mice at 14 dpl; *n*=3 animals for each genotype. *P*<0.001, Student's *t*-test. Data are presented as mean±s.e.m. **P<0.01, ***P<0.001; Student's *t*-test. Scale bars, 100 μm (**a**,**b**), 2 μm (**c**,**j**) and 50 μm (**f**,**g**).

In the developing spinal cord of *Tcf7l2*ΔHMG mice, expression of *Mbp* was substantially reduced at P0 and P7 (Fig. 2a), whereas the number of *PDGFR*α^+^ OPCs was comparable to controls at P7 and P14 (Supplementary Fig. 3). In adulthood at P60, however, *Mbp* expression was similar to control (Fig. 2b). Consistently, myelin ultrastructure, the percentage of myelinated axons and their *g*-ratios were all comparable between control and adult *Tcf7l2*ΔHMG spinal cord at P60 (Fig. 2c–e), indicating a delayed myelination process in the *Tcf7l2*-mutant spinal cord.

As myelination in the spinal cord fully caught up when the *Tcf7l2* mutants reached adulthood, we then capitalized on this phenotype to assess the function of Tcf7l2 in remyelination by employing the lysolecithin (LPC)-induced demyelination^26^. Local injection of LPC in the white matter induces rapid myelin breakdown and removal of myelin from adult CNS; myelin regenerates through an OPC recruitment phase at 7 days post lesion (dpl) and a remyelinating phase at 14 dpl^26^. In adult control mice, *Tcf7l2* was drastically upregulated at 7 and 14 dpl within the LPC lesions (Fig. 2f), consistent with the previous findings^8^. To determine whether *Tcf7l2*ΔHMG is required for myelin repair, we analysed myelin gene expression in the lesions at 14 dpl, a phase of OL regeneration and remyelination. Compared with controls, we detected substantially lower levels of *Mbp* and *Plp1* during remyelination in *Tcf7l2*ΔHMG mice (Fig. 2g,i), although PDGFRα^+^ OPCs were generated normally (Fig. 2g,h). Importantly, many fewer myelinated axons were detected in the lesions of *Tcf7l2*ΔHMG mice than in controls (Fig. 2j). The percentage and thickness of newly generated myelin sheaths around axons were significantly reduced in the *Tcf7l2* mutants (Fig. 2k,l). These observations indicate that Tcf7l2 activity is critical for remyelination after demyelinating injury.

### Downregulation of myelin genes in Tcf7l2ΔHMG mutants

**Figure 3:**
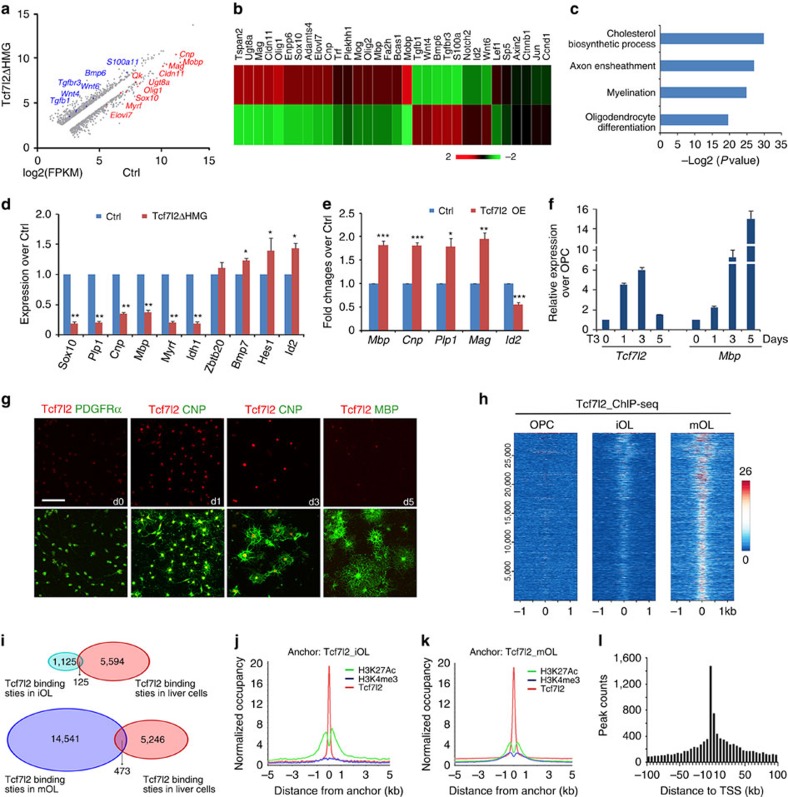
Transcriptome analysis of Tcf7l2-regulated genes and direct targets identified by ChIP-seq. (**a**) A representative scatter plot of RNA-seq (log2 scale) from control and *Tcf7l2*ΔHMG optic nerves at P12 (fold change>1.5). Downregulated genes are labelled in red colour and upregulated genes in blue colour. (**b**) Heatmap of representative altered gene expression in RNA-seq analysis of control and *Tcf7l2*ΔHMG optic nerves at P12. (**c**) GO analysis identified biological processes that involve genes significantly downregulated in *Tcf7l2*ΔHMG compared with control mice at P12. (**d**) qRT–PCR validation of downregulation of myelination-associated genes in *Tcf7l2*ΔHMG optic nerves compared with controls (*n*=three animals per genotype). (**e**) qRT–PCR analysis of expression of myelination-related genes in rat OPCs after transfection with control and the Tcf7l2-overexpressing (OE) vectors for 72 h; *n*=3 independent experiments. (**f**) Real-time qRT–PCR analysis of *Tcf7l2* and *Mbp* expression in OL lineage cells. OPCs were cultured under differentiation conditions in medium containing T3 for 0, 1, 3 and 5 days (*n*=3 independent treatments). (**g**) Immunostaining for Tcf7l2 expression in OPCs (PDGFRα^+^), differentiating and maturating OLs (CNP^+^), terminal differentiated OL (MBP^+^) induced by triiodothyronine (T3) treatment of OPCs for 0, 1, 3 and 5 days (d), respectively. (**h**) Heatmap of Tcf7l2-binding signals in OPCs (left), iOLs (OPC exposure to T3 for 1day, middle) and mOLs (OPC exposure to T3 for 3 days, right). Each line on the *y* axis represents a genomic region ±1.0 kb flanking Tcf7l2 summits. (**i**) The Venn diagram demonstrates minimal overlap of Tcf7l2 occupancy between iOLs or mOLs and H4IIE liver cells. ChIP-seq binding profiles of Tcf7l2 around H3K27Ac peak summits in iOLs (**j**) and mOLs (**k**). (**l**) The distribution pattern of Tcf7l2-binding regions in mOLs mapped to their closest TSS sites. Data are presented as mean±s.e.m. *P<0.05, **P<0.01 and ***P<0.001; Student's *t*-test. Scale bar, 50 μm (**g**).

In light of our data demonstrating impaired re/myelination capacity in the absence of Tcf7l2, we sought to identify the Tcf7l2-regulated genes. We carried out RNA-sequencing (RNA-seq) analysis using the OL-enriched optic nerves from control and *Tcf7l2*ΔHMG mice at P12, to identify differentially expressed genes. In accordance with dysmyelinating phenotypes, expression of myelin genes such as *Cnp*, *Mbp*, *Ugt8a* and *Mog*, and myelination-regulatory genes such as *Sox10*, *Olig1* and *Myrf*, was significantly reduced in *Tcf7l2* mutants (Fig. 3a,b). In contrast, we observed an upregulation of OL differentiation inhibitors, including *Id2*, *Notch2*, *Tgfb1/Tgfbr3* and *Bmp6*, as well as Wnt signalling pathway components including *Wnt4/6*, *Axin2*, *Ctnnb1*, *Ccnd1* and *Sp5* (Fig. 3a,b). Gene ontology analysis of downregulated genes identified cholesterol biosynthesis, axon ensheathment, OL differentiation and myelination (Fig. 3c), congruent with impaired OL differentiation in *Tcf7l2* mutants. We further confirmed the downregulation of these myelination-associated genes and differentiation regulators by qRT–PCR analysis (Fig. 3d). Moreover, overexpression of Tcf7l2 in OPCs enhanced expression of myelin genes such as *Mbp*, *Cnp*, *Plp1* and *Mag*, while repressing *Id2* expression (Fig. 3e). These observations suggest that Tcf7l2 transcriptional activity is both necessary and sufficient for OPC maturation.

### Stage-specific Tcf7l2 targeting for OL lineage progression

To determine the expression pattern of Tcf7l2 during OL lineage progression, we treated rat OPCs with triiodothyronine (T3) for different durations. *Tcf7l2* messenger RNA upregulated in differentiating OPCs after 1 and 3 days of T3-induced differentiation (Fig. 3f), but fell to a lower level in terminally differentiated OLs 5 days after T3 treatment. Similarly, Tcf7l2 immunoreactivity was weakly detected in PDGFRα^+^ OPCs but increased substantially in CNP^+^ differentiating OLs and then decreased in terminally differentiated MBP^+^ OLs (Fig. 3g).

To gain insights into the direct targets regulated by Tcf7l2, we carried out ChIP-seq analysis for Tcf7l2-chromatin occupancy in OPCs, immature OLs (iOL, OPC exposure to T3 for 1 day) and maturing OLs (mOLs, OPC exposure to T3 for 3 days)^27^ (Fig. 3h). The closest annotated gene to each Tcf7l2-binding site was identified as a presumed target. The number of Tcf7l2-targeted sites was ∼1,125 in iOLs and 14,541 in mOLs, respectively (Fig. 3i). The majority of Tcf7l2-binding peaks in iOLs overlapped with those identified in mOLs, but were of lower intensity (Fig. 3h). The increase in targeted sites and signal intensity from OPC to mOL correlated with the progression of OPC differentiation. In contrast, Tcf7l2-targeted sites in iOL and mOLs, respectively, overlapped by only 10 and 3% with those in other cell types such as H4IIE liver cells (Fig. 3i), suggesting that Tcf7l2 targets unique sets of genes in the OL lineage and possesses a distinct role in the control of OL differentiation.

The Tcf7l2-binding sites were co-localized with evolutionarily conserved enhancer elements marked by an activating histone mark H3K27ac^28^ in iOL and mOL cells (Fig. 3j,k). To evaluate the global distribution of Tcf7l2-binding loci, we plotted the number of Tcf7l2 sites against their distance to the nearest transcription start site (TSS). We detected a strong enrichment for Tcf7l2 binding around TSSs within 5-kb promoter regions of genes marked by the histone mark H3k4me3 (ref. 29), in particular in mOLs (Fig. 3l). These observations indicate that Tcf7l2 targets primarily to enhancer/promoter regions to regulate target gene expression.

### Wnt inhibitor Kaiso is a Tcf7l2 co-factor in iOLs

**Figure 4:**
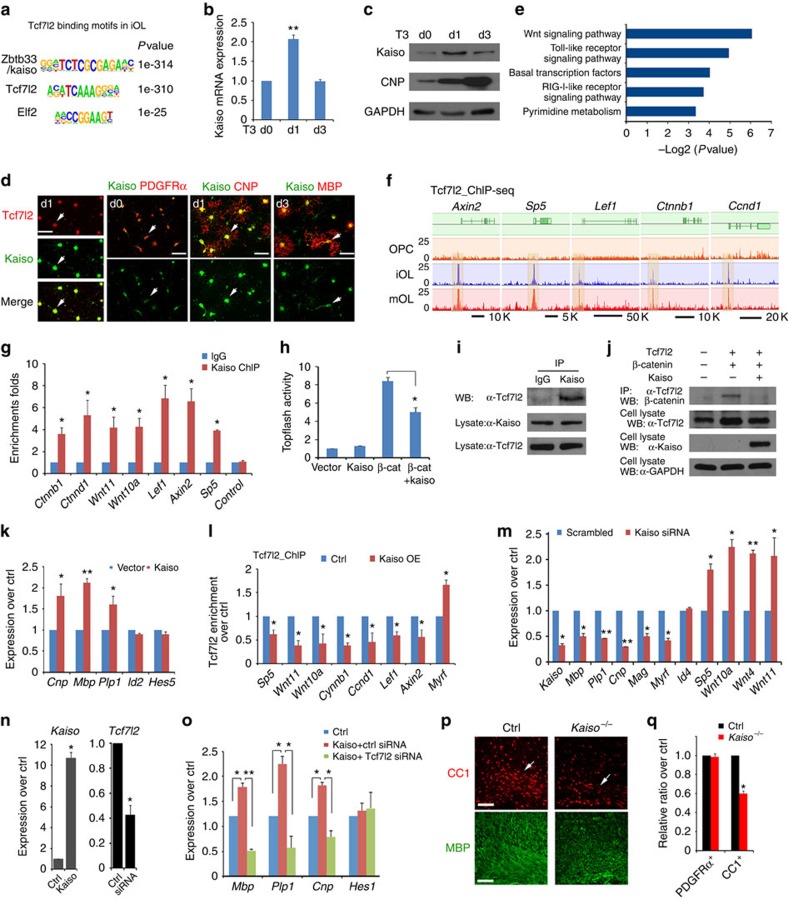
Tcf7l2 coordinates with Kaiso to inhibit Wnt/β-catenin signalling during OL differentiation. (**a**) Kaiso and Tcf7l2/Tcf4 consensus sites are most overrepresented in Tcf7l2-binding regions of iOLs. (**b**) qRT–PCR analysis of *Kaiso* in OPCs under T3-containing differentiation conditions for indicated days; *n*=3 independent experiments. (**c**) Kaiso and CNP expression at indicated days after T3 treatment of rat OPCs. (**d**) Co-expression of Kaiso with Tcf7l2, PDGFRα, CNP and MBP at indicated days after T3 treatment of OPCs. Arrows indicate co-labelled cells. (**e**) Biological processes overrepresented in Tcf7l2-occupied regions in iOLs. (**f**) Tcf7l2-binding profiles in OPCs, iOLs and mOLs on representative Wnt-signalling gene loci. (**g**) Kaiso occupancy on Tcf7l2-binding sites in iOLs by ChIP–qPCR. Control: genomic segment lacking Tcf7l2-binding sites. (**h**) Topflash luciferase activity in Hek293T cells transfected with expression vectors for Kaiso, β-catenin or both together. (**i**) Kaiso co-immunoprecipitated with Tcf7l2 in iOLs. (**j**) Co-immunoprecipitation with anti-Tcf7l2 from Hek293T cells transfected with Tcf7l2 with β-catenin and Kaiso for 48 h. Glyceraldehydes 3-phosphate dehydrogenase (GAPDH) as a loading control. (**k**) qRT–PCR analysis of myelination-related genes in OPCs transfected with control or Kaiso-expressing vectors for 48 h; *n*=3 independent experiments. (**l**) Tcf7l2 occupancy by ChIP–PCR in rat OPCs transfected with control or Kaiso-expressing vectors on the promoters of targeted genes; *n*=3 independent experiments. (**m**) qRT–PCR analysis of myelination-related and Wnt signalling genes in OPCs transfected with scrambled control and Kaiso siRNAs; *n*=3 independent experiments. Expression of *Kaiso* and *Tcf7l2* (**n**), as well as myelination-related genes (**o**) in Oli-neu cells transfected with control and Kaiso-overexpressing vectors and/or scrambled control and Tcf7l2-siRNAs; *n*=3 independent experiments. (**p**,**q**) The corpus callosum (arrows) of control and *Kaiso*^*−/−*^ mutants at P7 were immunostained with CC1 and MBP (**p**). Panel **q** depicts the percentage of PDGFRα^+^ and CC1^+^ cells in the corpus callosum at P7; *n*=3 animals per genotype. Data are presented as mean±s.e.m. *P<0.05, **P<0.01 and ***P<0.001; Student's *t*-test, except in **o** with analysis of variance (ANOVA) and Newman–Keuls multiple comparison test. Scale bars, 50 μm (**d**) and 100 μm (**p**,**q**).

To investigate whether certain DNA motifs were enriched in Tcf7l2-binding sites, we applied a motif-discovery algorithm, HOMER^30^. The sequence motif A(C/G)(A/T)TCAAAG identified in iOLs matches the consensus-binding motif for Tcf7l2 in previous ChIP-seq data sets (Fig. 4a)^31^,^32^. Enhancer regions typically have binding sites for co-factors, which bind within ∼100 bp of the Tcf7l2 peak summit. We found that a substantial proportion (∼28%) of Tcf7l2-binding sites were significantly overrepresented with the binding-motif of Zbtb33/Kaiso (Fig. 4a). Kaiso is a transcriptional repressor of Wnt signalling, it interacts with TCF factors to inhibit β-catenin-dependent activation of transcription^33^,^34^,^35^. Kaiso expression increased as OPC differentiated into iOL, but downregulated in mOL by qRT–PCR and western blot analyses (Fig. 4b,c). Consistently, by immunostaining, in contrast to weak expression in PDGFRα^+^ OPCs, Kaiso was upregulated and co-localized with Tcf7l2 in iOLs (Fig. 4d), while downregulated in MBP^+^ OLs, suggesting a potential role of Kaiso at the onset of OPC differentiation.

Gene ontology analysis indicated that Tcf7l2-targeted genes in iOL were significantly enriched in components of the Wnt signalling pathway (Fig. 4e). Tcf7l2 targeted to a number of the prototypical Wnt-responsive genes including *Axin2*, *Sp5*, *Lef1*, *Ctnnb1* and *Ccnd1* on the transition of OPCs to iOLs (Fig. 4f) and this was maintained in mOLs. We further confirmed that these loci were also co-occupied by Kaiso using ChIP–qPCR in iOLs (Fig. 4g). To examine the effects of Kaiso on Wnt signalling activity, we transfected Kaiso-expressing vectors together with constitutively active β-catenin (ΔN89 β-catenin)^36^ and a luciferase reporter for β-catenin/TCF activation, Topflash^37^, into HEK293T cells. Expression of ΔN89 β-catenin activated the Topflash reporter significantly; however, Kaiso expression suppressed the Topflash activity induced by the activated β-catenin (Fig. 4h). This suggests that Kaiso expression attenuates Wnt signalling activation, which negatively regulates OL differentiation^8^,^9^.

Co-immunoprecipitation revealed that endogenous Tcf7l2 and Kaiso were co-associated in the same complex in iOLs (Fig. 4i). To further investigate the functional interactions between Tcf7l2 and β-catenin or Kaiso, we performed co-immunoprecipitation in 293T cells transfected with expression constructs carrying Tcf7l2, β-catenin or Kaiso alone or in combination. Consistent with previous studies^17^, Tcf7l2 and β-catenin were detected in the same complex; however, in the presence of Kaiso, the interaction between Tcf7l2 and β-catenin was abolished (Fig. 4j). Similarly, overexpression of Kaiso substantially attenuated the interaction between endogenous Tcf7l2 and β-catenin in Oli-neu cells, an oligodendroglial cell line^38^ (Supplementary Fig. 4a). These observations suggest that Kaiso competes with β-catenin for Tcf7l2 binding and thereby inhibits Wnt/β-catenin signalling. Furthermore, overexpression of Kaiso in rat OPCs enhanced the expression of myelin-associated genes such as *Cnp*, *Mbp* and *Plp1* (Fig. 4k).

To determine the effect of Kaiso on the genome occupancy of Tcf7l2 on different target genes, we performed Tcf7l2 ChIP–qPCR in primary rat OPCs transfected with a *Kaiso*-expressing vector. We found that Tcf7l2 occupancy on the promoter of Wnt target genes (that is, *Sp5*, *Ctnnb1*, *Ccnd1*, *Wnt11* and *Wnt10a*) was reduced in Kaiso-overexpressing cells (Fig. 4l). In contrast, Tcf7l2 occupancy on the promoter of a myelination-promoting factor Myrf was enhanced (Fig. 4l), suggesting that Kaiso expression levels modulate the genome occupancy of Tcf7l2 in OPCs. To further examine the effect of *Kaiso* knockdown on the OL differentiation programme, small interfering RNA (siRNA) targeting *Kaiso* was transfected into rat OPCs and cultured under differentiation conditions for 72 h. *Kaiso* knockdown resulted in the downregulation of myelination-associated genes that included *Mbp*, *Plp1*, *Cnp*, *Mag* and *Myrf*, while upregulating the Wnt signalling effectors *Sp5*, *Wnt10a*, *Wnt4* and *Wnt11* (Fig. 4m), all as compared with scrambled siRNA-transfected OPCs. Furthermore, we found that Kaiso overexpression enhanced the expression levels of myelin-associated genes, while *Tcf7l2* knockdown blocked the ability of Kaiso to promote their expression (Fig. 4n,o), suggesting that the effect of Kaiso on enhancing OL differentiation gene expression depends on Tcf7l2. In addition, to determine the *in vivo* role of Kaiso in OL development, we analysed the phenotype of *Kaiso*-null mice^39^ and found that the number of CC1^+^ differentiating OLs and MBP signal intensity were substantially reduced in the corpus callosum of *Kaiso* mutants at P7 (Fig. 4p,q) compared with controls, despite normal PDGFRα^+^ OPC formation (Fig. 4q). Collectively, these data suggest that Kaiso promotes OPC differentiation programmes, while repressing Wnt signalling activity and is required for normal OL differentiation.

### Sox10 is a Tcf7l2 co-regulator for OL maturation

**Figure 5:**
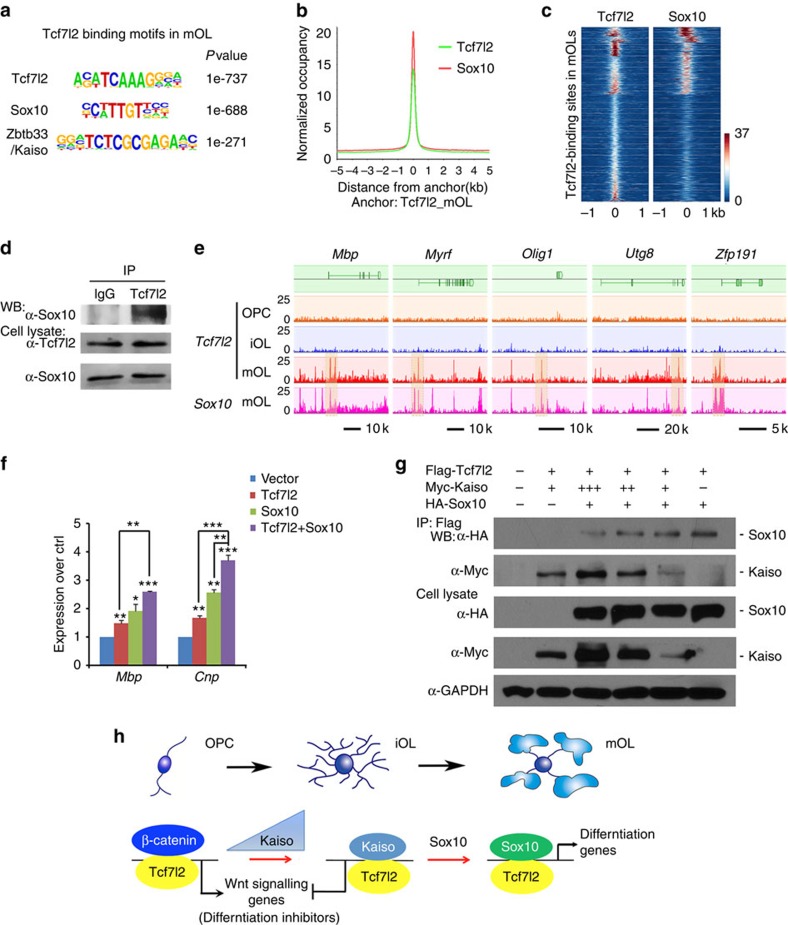
Tcf7l2 coordinates with Sox10 to regulate OL terminal differentiation. (**a**) *De novo* motif analysis identified Tcf7l2/Tcf4, Sox10-binding motifs as most significant binding motifs in Tcf7l2-binding regions in mOLs. (**b**) ChIP-seq binding profiles of Sox10 around Tcf7l2 peak summits in mOLs. (**c**) Heatmap of the signal intensities from ChIP-seq assays of Tcf7l2 and Sox10 across Tcf7l2-binding sites (±1 kb) called in mOLs. (**d**) Sox10 co-immunoprecipitated with Tcf7l2 in mOLs. (**e**) Visualization of Tcf7l2-binding profiles in OPCs, iOLs and mOLs on representative myelin gene loci (*Mbp*, *Myrf*, *Olig1*,*Ugt8* and *Zfp191*). Sox10/Tcf7l2 co-occupancy (highlighted) in mOLs was also shown. (**f**) qRT–PCR assay for *Mbp*, *Cnp* expression in OPCs transfected with expression vectors for Tcf7l2, Sox10 or both; *n*=3 independent experiments. (**g**) The expression vector carrying Flag-Tcf7l2 was co-transfected with HA-Sox10 and a varied amount of Myc-Kaiso in 293T cells for 48 h. Lysates were co-immunoprecipitated with anti-Flag-Tcf7l2 and subjected to western blot analysis. Glyceraldehy 3-phosphate dehydrogense (GAPDH) as a loading control. (**h**) Model of Tcf7l2 regulation of OL differentiation through sequential interactions with Kaiso and Sox10 to promote stepwise OL lineage differentiation. At the onset of OPC differentiation, Tcf7l2 binds Kaiso to inhibit Wnt signalling activation and subsequently associates with Sox10 to promote OL maturation. Data are presented as mean±s.e.m. *P<0.05, **P<0.01 and ***P<0.001; analysis of variance (ANOVA) with Newman–Keuls multiple comparison test.

As in iOLs, we found that Tcf7l2-binding peaks in mOLs match its consensus DNA-binding motif (Fig. 5a); however, ∼38% of Tcf7l2-binding sites present in mOLs were predominantly enriched with the Sox-consensus binding motif C(T/A)TTG(T/A)(T/A), which matches most significantly with an OL differentiation-promoting factor Sox10 motif^40^,^41^ in the HOMER-motif discovery programme (Fig. 5a), but to a lesser extent with the Kaiso-binding motif. To determine whether Sox10 and Tcf7l2 co-target to the same regulatory elements in mOL, we then performed genome-wide occupancy of Sox10 in mOL using ChIP-seq. We found that ∼44% of Tcf7l2 peak sets overlapped with those of Sox10 occupancy (Fig. 5b and Supplementary Fig. 5). Sox10 motifs were enriched in Tcf7l2-binding sites in mOLs (Fig. 5b,c), suggesting that Tcf7l2 and Sox10 co-target the same elements in mOLs. To further determine whether Tcf7l2 may co-associate with Sox10, we performed co-immunoprecipitation assays and showed that Tcf7l2 and Sox10 were present in the same complex in mOLs (Fig. 5d), suggesting that Sox10 is a co-factor of Tcf7l2 during OL maturation.

A large proportion of Tcf7l2/Sox10 co-occupancy was within the regulatory elements of myelination-associated genes including *Mbp*, *Myrf*, *Olig1*, *Utg8* and *Zfp191* in mOLs, but not in OPCs or iOLs (Fig. 5e). In addition, although Sox10 or Tcf7l2 expression in rat OPCs elevated the expression level of myelin-associated genes such as *Mbp* and *Cnp*, co-expression of Sox10 and Tcf7l2 further increased their levels (Fig. 5f), suggesting that Sox10 and Tcf7l2 cooperate to promote myelin gene expression.

How does Tcf7l2 coordinate distinct co-factors to control OL lineage progression? Kaiso expression increases in iOL, but downregulates in mOL, while the Sox10 expression level is maintained throughout the OL lineage. To determine whether the temporal sequence of Tcf7l2 recruitment of Kaiso and Sox10 depends on their expression levels, we co-transfected Tcf7l2 with a varied amount of Kaiso, while keeping the Sox10 level constant in 293T cells. We found that Tcf7l2 was associated preferentially with Kaiso when Kaiso expression levels were high (Fig. 5g). As the Kaiso level fell, Tcf7l2 was found to interact with Sox10 (Fig. 5g). Furthermore, we found that overexpression of Sox10 substantially reduced association of Tcf7l2 with Kaiso in Oli-neu cells (Supplementary Fig. 4b), suggesting dosage-dependent competitive binding of Tcf7l2 with Kaiso and Sox10. Thus, there appears to be a two-step recruitment process in which Tcf7l2 association with ‘early-' and ‘late-binding' transcription factors, Kaiso and Sox10, respectively, is responsible for OL lineage progression. Together, these observations suggest that Tcf7l2 coordinates the stepwise OL differentiation process through interacting with Kaiso to suppress inhibitory Wnt signalling in OPCs, while associating with Sox10 to promote myelination-associated programmes during OL maturation (Fig. 5h).

### Tcf7l2 activates cholesterol biosynthesis for OL maturation

**Figure 6:**
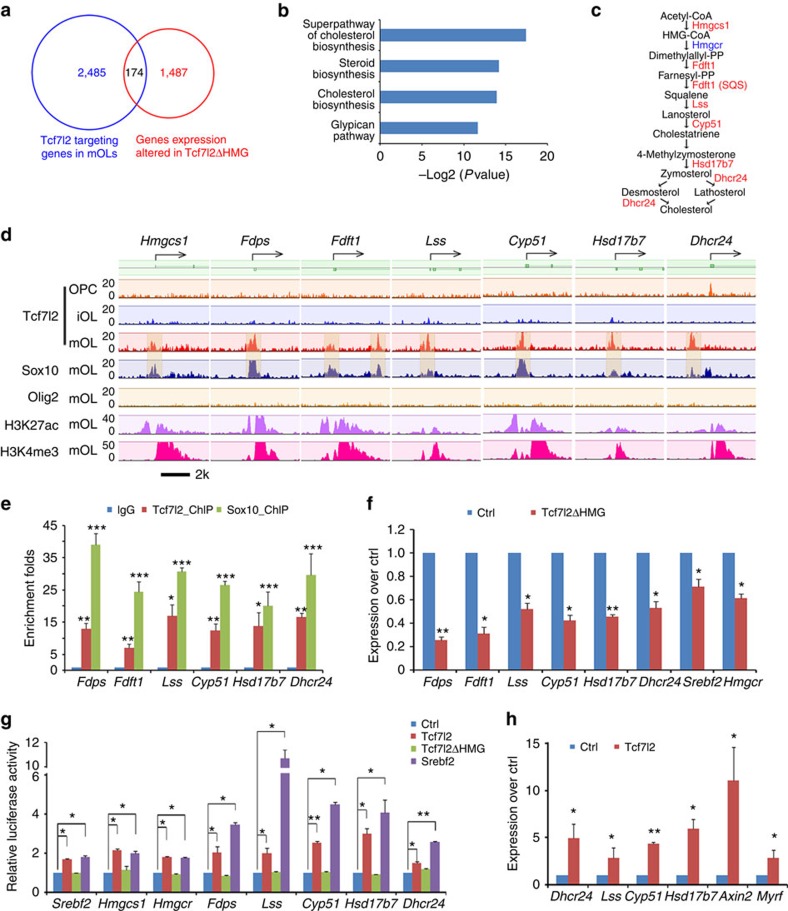
Tcf7l2 regulates the expression of cholesterol biosynthetic genes in OLs. (**a**) Venn diagram shows the overlap between Tcf7l2-targeting genes and substantially altered genes detected by RNA-seq in Tcf7l2ΔHMG optic nerves (fold change>1.5 between Ctrl and mutant). (**b**) GO analysis of pathways overrepresented by direct Tcf7l2 target genes in mOLs. (**c**) Schematic view of *de novo* cholesterol biosynthetic pathway; the putative direct Tcf7l2 target genes are highlighted in red. (**d**) Genome browser view of the distribution of Tcf7l2, Sox10, Olig2, H3K27ac and H3K4me3 binding to promoter regions of cholesterol biosynthesis genes (*Fdps*, *Fdft1*, *Lss*, *Cyp51*, *Hsd17b7* and *Dhcr24*) in OPCs, iOLs and mOLs as indicated. (**e**) ChIP–PCR assay for the enrichment of Tcf7l2 and Sox10 binding on the promoters of cholesterol biosynthesis genes over IgG controls; *n*=3 independent assays. (**f**) qRT–PCR analysis of the expression of cholesterol biosynthetic genes in *Tcf7l2*ΔHMG optic nerves versus controls; *n*=three animals per genotype. (**g**) Luciferase reporter activity driven by Tcf7l2-binding promoter/enhancer regions was assessed in 293T cells co-transfected with control and pcDNA3 expression vectors for Tcf7l2 or ΔHMG mutant Tcf7l2, or Srebf2; *n*=3 independent experiments. (**h**) qRT–PCR assay for the expression of cholesterol biosynthetic genes in OPCs transfected with the pcDNA3 expression vectors for control and Tcf7l2; *n*=3 independent experiments. Data are presented as mean±s.e.m. *P<0.05, **P<0.01 and ***P<0.001; Student's *t*-test, except in **g** with analysis of variance (ANOVA) and Newman–Keuls multiple comparison test.

Superimposing Tcf7l2 ChIP-seq from mOLs and RNA-seq data revealed that ∼174 targeted genes with substantial changes of expression in *Tcf7l2*ΔHMG mutants also exhibited strong Tcf7l2 binding to their proximal promoter regions (Fig. 6a). These genes were overrepresented in the functional categories of steroid biosynthesis and cholesterol metabolism (Fig. 6b). Among these Tcf7l2 targets in mOLs, we identified a cohort of genes that encode enzymes involved in *de novo* cholesterol biosynthesis, including *hmgcs1*, *Fdps*, *Fdft1*, *Lss*, *Cyp51*, *Hsd17b7* and *Dhcr24* (Fig. 6c,d). Although Tcf7l2 did not appear to directly target the gene locus of *Hmgcr* (Supplementary Fig. 6a), it was highly enriched on the promoter region of *Srebf2* (Supplementary Fig. 6b), a key upstream regulator of *Hmgcr* expression^42^,^43^.

When comparing Tcf7l2 occupancy on the promoters of cholesterol pathway genes with that of Olig2, an OL specification factor, we found that Tcf7l2 targeting was substantially enriched in parallel with OL maturation, and that these promoters were marked with the activating histone marks H3K4me3 and H3K27Ac^28^,^29^ (Fig. 6d). In contrast, Olig2 binding on these promoter regions was barely detectable in mOLs (Fig. 6d), suggesting a distinct role between Olig2 and Tcf7l2 in the differentiation of mOLs.

We next asked whether Tcf7l2 acted with partners to support oligodendrocytic cholesterol synthesis and found that in mOLs, Tcf7l2 co-occupied the promoters of cholesterol pathway genes with Sox10 (Fig. 6d); ChIP–qPCR further confirmed the enrichment of Tcf7l2 and Sox10 binding on the promoter elements of these cholesterol synthetic genes (Fig. 6e). The co-occupancy of the gene loci by Tcf7l2 and Sox10 is consistent with observations that Tcf7l2 and Sox10 form a complex in mOL.

The presence of H3K4me3 and H3K27Ac in the targeted promoters, both of which are indicators of active transcription state^28^,^29^, suggests that Tcf7l2 positively regulates expression of cholesterol biosynthesis genes (Fig. 6d). Accordingly, the mRNA expression levels of these Tcf7l2-targeted cholesterol biosynthesis genes was significantly reduced in *Tcf7l2*ΔHMG mutant optic nerves (Fig. 6f).

To compare the activity of Tcf7l2 with a key regulator for the cholesterol biosynthesis Srebf2/Srebp2 on target gene expression, we cloned the promoter regions carrying Tcf7l2-binding sites of cholesterol biosynthesis genes including *Srebf2*, *Hmgcs1*, *Hmgcr*, *Fdps*, *Lss*, *Cyp51*, *Hsd17b7* and *Dhcr24* into a luciferase reporter system. We found that similar to Srebf2, Tcf7l2 stimulated the luciferase reporter activity driven by these regulatory elements/enhancers (Fig. 6g). In contrast, these effects could not be observed with Tcf7l2ΔHMG (Fig. 6g).

To further explore the relative contribution of Tcf7l2 to expression of cholesterol biosynthesis genes in OLs, we next transfected purified OPCs with control and expression vectors carrying Tcf7l2 and found that overexpression of Tcf7l2 induced the expression of a number of genes encoding essential enzymes for cholesterol synthesis, including *Lss*, *Cyp51*, *Hsd17b7* and *Dhcr24* (Fig. 6h), as well as *Axin2*, a generic Wnt/Tcf7l2 target gene, and the myelination-promoting gene *Myrf*, suggesting that activation of Tcf7l2 promotes expression of cholesterol biosynthesis genes for OL differentiation.

### Tcf7l2-induced cholesterol synthesis for OL differentiation

**Figure 7:**
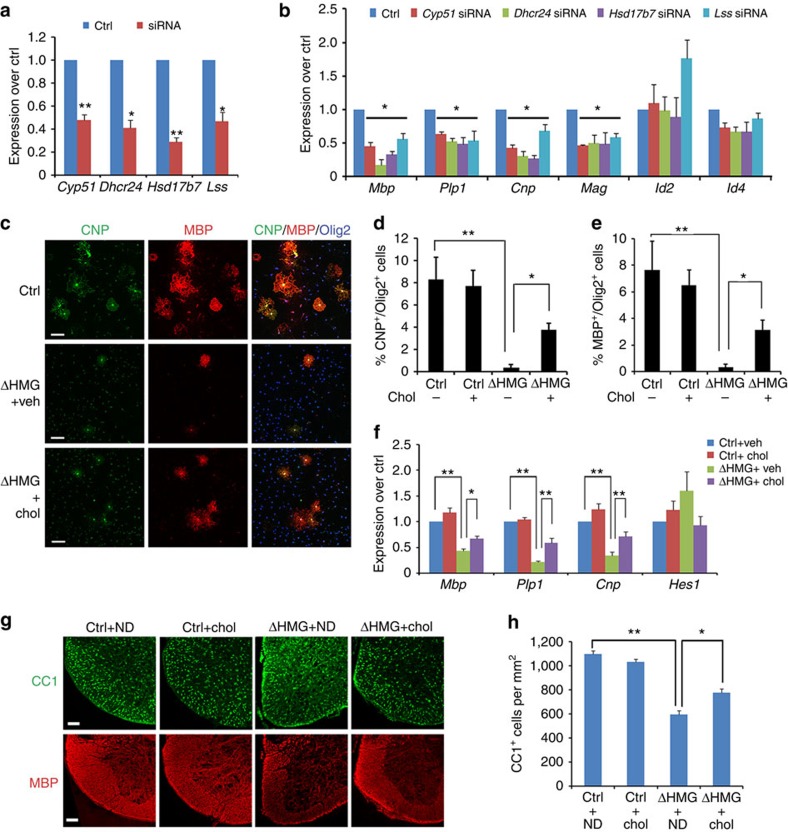
Induction of cholesterol biosynthetic pathway by Tcf7l2 is required for OL differentiation. (**a**) Inhibition of cholesterol biosynthetic genes in OPCs transfected with scrambled control siRNAs or siRNAs against *Lss*, *Cyp51*, *Hsd17b7* and *Dhcr24*. (**b**) Downregulation of expression of myelin genes in OLs transfected with *Cyp51*, *Lss*, *Dhcr24* and *Hsd17b7* siRNAs; *n*=3 independent experiments. (**c**) OPCs isolated from control and *Tcf7l2*ΔHMG animals were treated with vehicle (Veh) and cholesterol (Chol) in culture for 4 days in OPC growth medium without PDGF-AA. Expression of CNP (green), MBP (red) and Olig2 (blue) were examined by immunostaining. Quantification of the percentage of CNP^+^ (**d**) and MBP^+^ (**e**) OL cells among Olig2^+^ cells in control and *Tcf7l2*ΔHMG cells treated with vehicle or cholesterol; *n*=three independent experiments. (**f**) qRT–PCR assay for the expression of myelin-associated genes in control and *Tcf7l2*ΔHMG OPCs treated with vehicle or cholesterol (Chol) for 4 days; *n*=3 independent experiments. (**g**,**h**) Pregnant female mice were fed with normal diet (ND) or 2% cholesterol diets at the time of gestation. The spinal cords of control and *Tcf7l2*ΔHMG pups were harvested at P14 and immunostained with CC1 (green) and MBP (red). Representative images were shown in **g**. Scale bar, 100 μm. The number of CC1^+^ OLs per area (1 mm^2^) were quantified in the spinal cord of control and *Tcf7l2*ΔHMG mutants (**h**); *n*=3 independent animals. Data are presented as mean±s.e.m. *P<0.05, **P<0.01 and ***P<0.001; Student's *t*-test in **a**. Analysis of variance (ANOVA) with Newman–Keuls multiple comparison test in **d**–**f** and **h**. Scale bar, 50 μm (**c**).

Cholesterol biosynthesis is required for myelin sheath outgrowth as shown in *Hmgcs1* mutant zebrafish^44^ and *Fdft1* mutant mice^45^. To determine the role of other Tcf7l2-regulated cholesterol biosynthesis genes in OL differentiation, we carried out siRNA knockdown during OPC differentiation. Knockdown of *Cyp51*, *Dhcr24*, *Hsd17b7* and *Lss* (Fig. 7a) reduced expression of myelin genes including *Mbp*, *Plp1*, *Cnp* and *Mag*, although *Id2* and *Id4* expression was not significantly altered (Fig. 7b).

As Tcf7l2 activated the expression of cholesterol biosynthesis genes, we hypothesized that dysregulation of cholesterol synthesis might account for the impairment of OL maturation in *Tcf7l2*ΔHMG mice. Previous studies reported that cholesterol, a rate-limiting lipid component for myelin sheath growth, is not imported into the brain from the circulation but rather synthesized locally by myelin-forming OLs^46^,^47^. On that basis, we next asked whether exogenous cholesterol would restore the differentiation capacity of OPCs isolated from *Tcf7l2* mutants. Under differentiation conditions, control OPCs readily differentiated into CNP^+^ and MBP^+^ mature OLs, whereas the majority of *Tcf7l2*-mutant OPCs failed to mature into OLs with elaborate membrane processes (Fig. 7c). In addition, the proportion of CNP^+^ or MBP^+^ OLs was significantly reduced in *Tcf7l2* mutants compared with controls (Fig. 7d,e). Strikingly, exogenous cholesterol partially restored the percentage of CNP^+^ and MBP^+^ OLs in mutant cultures (Fig. 7c–e). Consistently, expression of myelin-associated genes increased in *Tcf7l2*-mutant OPCs treated with cholesterol (Fig. 7f). To further determine whether cholesterol supplementation could rescue the OL differentiation defect in *Tcf7l2*-mutant animals, we fed the pregnant mice carrying control and *Tcf7l2*ΔHMG mutants with cholesterol-enriched diet during gestation, to allow cholesterol intake into the CNS. The pups were harvested at P14 for analysis. Although cholesterol supplementation did not alter the number of OLs in the spinal cords of control animals, it significantly increased the number of CC1^+^ OLs and expression of MBP in *Tcf7l2* mutants (Fig. 7g,h). These observations suggest that cholesterol is responsible, at least in part, for Tcf7l2-dependent control of OL differentiation, and that Tcf7l2 directly activates the genes encoding cholesterol biosynthesis enzymes to promote OL differentiation and maturation.

## Discussion

Wnt/β-catenin/Tcf7l2 signalling has been suggested to positively or negatively regulate OL development, which may depend on the context^8^,^9^,^48^,^49^,^50^,^51^. Identification of Tcf7l2 direct targets and its co-regulators is of vital importance to understand the Tcf7l2-mediated process for OL development. Using integrative unbiased genomics analyses of multiple stages in the OL lineage, we present the first genome occupancy mapping of stage-specific targets of Tcf7l2 during OL lineage progression and further identify Tcf7l2-engaging partners that control transcriptional switches at the transitions of differentiation initiation and OL maturation. Strikingly, we uncover a non-canonical Wnt signalling co-repressor Kaiso as a Tcf7l2 partner at the OL differentiation onset to inhibit β-catenin activity. During OL maturation, Tcf7l2 further recruits another partner Sox10, a differentiation-promoting factor, to activate the myelinogenic transcriptional programme. Thus, Tcf712 executes a dual regulatory control by recruiting stage-specific partners to switch its functions to propel stepwise OL differentiation. Importantly, our studies further provide evidence that Tcf7l2 directly targets and activates cholesterol metabolism-associated genes and thereby regulates *de novo* cholesterol biosynthesis necessary for myelinogenesis, pointing to a previously unappreciated role of Tcf7l2 for cholesterol homeostatic control of CNS myelination.

Constitutive activation of Wnt/β-catenin signalling inhibits OL differentiation, suggesting that Wnt signalling may need to be attenuated for developmental OL differentiation. We found that the association of Kaiso with Tcf7l2 reverses the role of the β-catenin/Tcf7l2-mediated transcriptional complex from a differentiation repressor to an activator, which then promotes the transition of OPCs from an undifferentiated to differentiating state. At the onset of OPC differentiation, Kaiso is upregulated and appears to act as a competitive inhibitor of β-catenin, to oppose Wnt signalling activation by disrupting the β-catenin/Tcf7l2 complex. Kaiso has been shown to interact with the NCoR/Hdac1 complex to repress target gene expression^35^,^52^. It is possible that Tcf7l2 forms a potent repressive complex with Kaiso and Hdac1/2, and perhaps Hdac-associated Groucho/TLE repressors as well^9^,^53^, so as to inhibit Wnt/β-catenin signalling.

Our data indicate that Tcf7l2 targets a number of prototypical Wnt-responsive genes, including *Axin2*, *Ccnd1*, *Ctnnb1*, *Lef1* and *Sp5* on OPC differentiation into iOLs and persists with maturation (Fig. 4f). This suggests that Tcf7l2 may continue to modulate β-catenin activation and downstream target expression during OL differentiation. Persistent control of Wnt target gene expression and signalling by Tcf7l2 and its co-factors probably generates a high degree of regulatory complexity in response to different Wnt signalling inputs over the course of OPC differentiation.

We found that the transcriptional activity of Tcf7l2 is critical for OL differentiation and myelin repair. Although we could not rule out a potential dominant-negative effect of the Tcf7l2 mutant, the *Tcf7l2*ΔHMG mutant mice yielded a dysmyelinating phenotype similar to that of full-length *Tcf7l2*-deletion mutants^18^. Of note, we did not observe any discernable phenotype in *Tcf7l2*ΔHMG heterozygous mice. It thus seems unlikely that any dominant-negative effect would significantly contribute to the dysmyelinating phenotype. Although *Tcf7l2*ΔHMG was able to interact with Kaiso and Sox10 when overexpressed in 293T cells (Supplementary Fig. 7a,b), it could not activate myelin gene expression as wild-type Tcf7l2 (Supplementary Fig. 7c), but rather blocks Kaiso and Sox10 activity for myelin gene activation (Supplementary Fig. 7d,e). This suggests that Tcf7l2 activity is required for OL differentiation-promoting machinery. Tcf7l2ΔHMG mutant protein appears at a lower level in the *Tcf7l2*-mutant spinal cord compared with its wild-type counterpart (Fig. 1b). This is probably due to the severe reduction of differentiating OLs, where Tcf7l2 is highly expressed, in the mutants.

During OL maturation, we detect stronger signal intensity and an increase in Tcf7l2-targeted binding sites, which largely overlap with those identified in rat spinal cord^54^ (Supplementary Fig. 8). We find that Sox10 acts a co-regulator with Tcf7l2 in mOLs, and that Tcf7l2 and Sox10 cooperate to promote myelinogenic programmes, consistent with the notion that Tcf7l2 has a β-catenin independent function during OL differentiation^18^. These findings suggest that Tcf7l2 functions through the sequential operation of two interlaced gene regulatory networks: one blocking inhibitory Wnt signalling activity at iOL stages through recruiting transcriptional repressors such as Kaiso, and the other promoting a ‘terminal' differentiation process in committed OLs in cooperation with Sox10. The switch of Tcf7l2 co-regulators from Kaiso to Sox10 while transitioning from iOL to mOLs may lead to the further induction of myelin-specific genes, thus facilitating OL maturation (Fig. 5h). Cooperation with stage-specific, transiently expressed transcriptional partners may also explain the distinct stage-specific role of Tcf7l2 in the initiation and maintenance of OL differentiation.

Besides the temporally dynamic nature of Tcf7l2 interactions with other transcriptional modulators, we have also observed a distinct role for Tcf7l2 in regulating the extent of myelination. In contrast to persistent myelination defects in the brain of *Tcf7l2* mutants, Tcf7l2 appears dispensable for myelination in the adult spinal cord, where Tcf7l2 is hardly detectable. At present, the underlying mechanisms for this regional specificity remain undetermined. The efficient *Tcf7l2* allele recombination in the spinal cord (Fig. 1b) indicates that a region-specific function of Tcf7l2 rather than incomplete recombination accounts for the phenotypic difference.

A novel finding of our present study is that Tcf7l2 directly regulates *de novo* cholesterol biosynthesis and metabolism. We find that Tcf7l2 target genes are selectively enriched on the promoter regions of genes involved in cholesterol biosynthesis in mOLs. In contrast, in liver cells, Tcf7l2 targets are enriched on the promoter/enhancers of classical lipid synthesis-related genes such as *Fabp1*, *Apod*, *Scd1*, *Mttp*, *Hnf4a* and *Fas*, which are associated primarily with lipid metabolic pathways, such as the synthesis of ketone bodies, transport of fatty acids and gluconeogenesis^32^,^55^ (Supplementary Fig. 9). This suggests a conserved yet distinct role for Tcf7l2 in the control of lipid biosynthesis and metabolism, by regulating different sets of genes in a cell-type-specific manner.

Cholesterol is a rate-limiting lipid component for OL myelination and myelin sheath growth^46^,^47^. Defects in myelination has been observed in mutants in the genes responsible for cholesterol biosynthesis such as in *Fdft1* mutant mice^45^ and in *Hmgcs1* mutant zebrafish^44^. We find that cholesterol supplementation could, at least in part, rescue OL differentiation defects in *Tcf7l2*ΔHMG mutants. Interestingly, a similar observation was recently reported that cholesterol injection could rescue the axon wrapping defect in *hmgcs1* mutant zebrafish, suggesting that cholesterol synthesis is necessary for OL maturation and axon wrapping^44^. A cell sensing insufficient cholesterol could block OL differentiation and expression of myelination-associated genes. It is possible that a negative feedback or ‘check point' mechanism exists, whereby sufficient cholesterol biosynthesis for myelin biosynthesis must be verified before transcription of myelination-promoting factors. Given that Tcf7l2 mutations lead to downregulation in the expression of cholesterol biosynthesis enzymes, the requirement of Tcf7l2 for OL myelination can be mediated, at least partially, through its role as a regulator of cholesterol biosynthesis during OL maturation. Collectively, Tcf7l2 may have a parallel role in regulating the expression of cholesterol biosynthesis genes in addition to directly activating the myelinogenic transcriptional programme.

Previous studies show that TCF7L2 expression rose during active remyelination in both human patients and rodent models^8^,^56^, and yet is completely absent in the lesions of chronic multiple sclerosis^57^. This observation suggested the potential requirement of TCF7L2 for active myelin repair. Here we provide evidence that Tcf7l2 transcriptional activity is crucial for remyelination after demyelination, as well as to normal myelin formation during brain development. Indeed, as the impact of the *Tcf7l2* knockout on myelination is more severe in the brain than in the spinal cord, it would seem likely to be that the remyelination defects observed in the *Tcf7l2*ΔHMG spinal cord should manifest in the brain as well.

Recent studies indicate that TCF7L2 is a key regulator of the metabolic gene programme that controls transcriptional responses to metabolic challenge in the liver, muscle or white adipose tissues^32^. For instance, *TCF7L2* single-nucleotide polymorphism variants and mutations contribute to type 2 diabetes mellitus^58^,^59^,^60^. Yet, type 2 diabetes impairs OL regeneration in the white matter lesions after ischaemic injury^61^. Our findings suggest then that TCF7L2 mutations or single-nucleotide polymorphism variants might modify both the degree of ischaemic myelin damage in patients with type 2 diabetes and their ability to remyelinate. Together, integrative analyses of Tcf7l2 occupancy, gene expression profiling and binding motifs at multiple OL stages reveal that Tcf7l2 appears to exercise its functions by cooperating with stage-specific co-regulators to control OL differentiation programmes. More broadly, the present genome-wide Tcf7l2 target identification should provide us a framework to identify novel therapeutic avenues for myelin repair in the CNS.

## Methods

### Generation of Tcf7l2 conditional knockout mice

*Tcf7l2* floxed mice^20^ were crossed with *Olig1*-Cre^22^, *Olig2*-Cre^62^ (Olig2<tm2(TVA,cre)Rth>/J from Jackson Laboratory) and *CNP*-Cre^25^ (gift of Dr Klaus-Armin Nave) mice to generate *Tcf7l2*ΔHMG (*Tcf7l2*^fl/fl^:*CNP*-Cre^+/−^) and heterozygous control (*Tcf7l2*^fl/+^:*CNP*-Cre^+/−^) mice. As LoxP sites flank exon 11 encoding the highly conserved DNA-binding domain of Tcf7l2, Cre-mediated recombination results in Tcf7l2 mutant protein lacking DNA-binding transcriptional activity, while the mutant mRNA and protein may still be expressed. The control mice developed and behaved the same as wild type. PDGFRα–GFP (Jackson Laboratory Stock Number 024098) and *CNP*-mGFP reporter mice^23^, and Zbtb33/Kaiso mutant mice^39^ were used for phenotype analysis. We used both male and female mice for the study. The mouse strains used in this study were generated and maintained on a mixed C57Bl/6;129Sv;CD-1 background. All animal use and studies were approved by ethical committees of our institutions and by the Institutional Animal Care and Use Committee of Cincinnati Children's Hospital Medical Center, USA.

### Tissue processing and histochemistry

CNS tissues were dissected and fixed overnight in 4% (w/v) paraformaldehyde (PFA) and processed for cryosectioning or paraffin embedding and sectioning. The tissue processing and immunohistochemical staining procedures were performed as described previously^27^. Briefly, for tissue immunostaining, cryosections were incubated overnight in primary antibodies diluted in block solution (PBS with 5% v/v normal goat serum (Sigma-Aldrich, St Louis) and 0.3% v/v Triton X-100). After washing with PBS, sections were then incubated overnight at 4 °C with corresponding Cy2 or Cy3 fluorophore-conjugated secondary antibodies (Jackson ImmunoResearch). For BrdU staining, cells or tissue sections were denatured with 0.1 N HCl for 1 h in a 37 °C water bath. After denaturation, sections were neutralized with 0.1 M Borax pH 8.5 (Sigma) for 10 min. Sections were washed with 0.3% Triton X-100/1 × PBS (wash buffer) for three times and blocked with 5% normal donkey serum (Sigma-Aldrich) containing wash buffer for 1 h at room temperature. Mouse anti-BrdU (BD Bioscience, 550891, 1:500) antibody was used to label BrdU overnight at 4 °C. Samples were mounted in Fluoromount G (SouthernBiotech) for fluorescent microscopy. For BrdU incorporation analysis, control and *Tcf7l2*ΔHMG littermates were injected with BrdU (Sigma-Aldrich) (100 mg kg^−1^ body weight) 2 hr before killing. Primary antibodies used were as follows: Olig2 (Millipore, AB9610, 1:1,000), BrdU (BD Bioscience, 550891, 1:500), PDGFRα (BD Bioscience, 558774, 1:500), CC1 (Calbiochem, OP80, 1:500), NeuN (Millipore; MAB377, 1:500), GFAP (Sigma, G3893, 1:500), MBP (Santa Cruz; sc-13914, 1:500), Sox10 (Santa Cruz, sc-17343, 1:300); Tcf7l2 (Cell Signaling Technology, #2565, 1:500), FLAG (Cell Signaling Technology, #2368, 1:500), Myc (Santa Cruz, sc-789, 1:500), MAG (Cell Signaling Technology, #9043, 1:500), Iba1 (Waco; 019-19741, 1:400) and Kaiso (Abcam, ab12723, 1:300). The Kaiso antibody was validated with vectors expressing Kaiso and Myc-tag Kaiso-transfected 293T cells by western blotting and immunostaining (Supplementary Fig. 10a,b). In addition, Kaiso expression was abolished in the brain of *Kaiso*-null mice by western blot analysis (Supplementary Fig. 10c), confirming the antibody specificity.

RNA *in situ* hybridization was performed using digoxigenin-labelled riboprobes as described previously^63^. The probes used were as follows: murine *PDGFRα*, *Plp1/Dm-20* and *Mbp*.

### Oligodendroglial cell culture and transfection

Isolation of primary rat OPCs from cortices of P2 pups was performed as previously described^64^. OPCs were differentiated in OL differentiation medium (Sato medium supplemented with 15 nM T3 and 10 ng ml^−1^ ciliary neurotrophic factor) for 24 and 72 h to become iOL and mOL as the initiation and maturing phases of OLs, respectively, as previously described^27^. Mouse OPCs were isolated from P5 to P7 cortices of control and *Tcf7l2*ΔHMG mutants by immunopanning with antibodies Ran-2, GalC and PDGFRα sequentially as previously described^65^. The mouse OL cell line Oli-neu cells were maintained in proliferation medium consisting of DME/F12 medium supplemented with Sato, NT3, CNTF, B27, 0.5 μM T3, 0.5 μM T4 and 1% horse serum. The cells were induced to differentiate with 1 mM cyclic AMP supplementation^38^.

Primary rat OPCs or Oli-neu cells were transfected with control and corresponding expression vectors carrying Tcf7l2, Tcf7l2ΔHMG and Kaiso, or siRNAs by using Nucleofector (Lonza) according to the manufacturer's protocol. The cells were harvested 72 h after transfection and processed for qRT–PCR or western blot analysis. siRNAs were purchased from Sigma-Aldrich with the following catalogue numbers: for *Cyp51* (SASI_Rn02_00342897), *Dhcr24* (SASI_Rn02_00228595, SASI_Rn02_00228596, SASI_Rn02_00228597), *Lss* (SASI_Rn01_00111479, SASI_Rn01_00111481, SASI_Rn01_00111484), *Hsd17b7* (SASI_Rn02_00242485, SASI_Rn02_00242486, SASI_Rn02_00242487), *Kaiso* (SASI_Rn02_00247204, SASI_Rn02_00247205, SASI_Rn02_00247206) and *Tcf7l2* (SASI_Mm01_00142189, SASI_Mm02_00315891, SASI_Mm01_00142191).

### Co-immunoprecipitation and luciferase assays

Co-immunoprecipitation and western blotting were performed as described previously^27^. Briefly, for co-immunoprecipitation in iOLs and mOLs, 600 μg of cell lysate proteins were incubated with 3 μg anti-Tcf7l2, anti-Kaiso or anti-Sox10 for immunoprecipitation assay. For Tcf7l2/β-catenin complex competition assay, in Oli-neu cells, cells were transfected with 3 μg each pCS2-Myc-Kaiso or pcDNA3-HA-Sox10; in HEK293T cells, cells were transfected with pcDNA3 Flag-tag Tcf7l2, β-catenin, Myc-tag Kaiso or pcDNA3-HA-Sox10. For luciferase assays, HEK293T cells were transiently transfected with pCS2-Myc-tag Kaiso and/or β-catenin^9^, together with a Topflash reporter, or transfected with pcDNA3-Flag-Tcf7l2 or Srebf2 with individual luciferase reporters driven by the enhancers of cholesterol biosynthesis genes. After western blotting, proteins were detected with appreciate secondary antibodies by using chemiluminescence with the ECL kit (Pierce) according to the instructions of the manufacturer. The primers used for cloning of cholesterol biosynthesis gene promoters are listed in Supplementary Table 1. Western blotting images have been cropped for presentation. Full-size images for the main figures and for the Supplementary Figs are presented in Supplementary Figs 11 and 12, respectively.

### Quantitative real-time PCR analysis

RNAs were isolated with Trizol (Invitrogen Inc.) from cells or snap-frozen tissues. Reverse transcription was performed with the cDNA Reverse Transcription Kit (Bio-Rad) with iQ SYBR Green Supermix (170-8880). qRT–PCR was carried out using the ABI Prism 7900 Sequence Detector System (Perkin-Elmer Applied Biosystems) using *Gapdh* as an internal control. Each analysis was performed in triplicates and the results were normalized to *Gapdh* for each sample. The qRT–PCR primer sequences are listed in Supplementary Table 1.

### RNA-seq and data analysis

We isolated RNAs from the optic nerves of control mice and *Tcf7l2*ΔHMG mutants at P12 and subjected samples to RNA deep sequencing and data analysis as previously described^27^. RNA-seq libraries were prepared using Illumina RNA-seq Preparation Kit (Illumina) and sequenced on a HiSeq 2000 sequencer. RNA-seq reads were mapped using TopHat with default settings (http://tophat.cbcb.umd.edu). TopHat output data were then analysed by Cufflinks to (1) calculate fragments per kilobase of transcript per million mapped reads values for known transcripts in mouse genome reference and (2) test the changes of gene expression between *Tcf7l2*ΔHMG and control. Heatmap of gene differential expression was generated using R language (http://www.r-project.org).

### ChIP-sequencing and ChIP–qPCR

ChIP assays were performed as previously described, with minor modifications^27^. Briefly, OPCs, iOLs and mOLs (∼20 million cells) were fixed for 10 min at room temperature with 1% formaldehyde-containing medium. Nuclei were isolated and sonicated in sonication buffer (10 mM Tris-HCl pH 8.0, 1 mM EDTA, 0.5 mM EGTA and protease inhibitor cocktail). Sonicated chromatin (∼300 μg) was used for immunoprecipitation by incubation with appropriate antibodies (4 μg) overnight at 4 °C. Ten per cent of chromatin used for each ChIP reaction was kept as input DNA. Prerinsed magnetic protein A/G beads (50 μl) were added to each ChIP reaction and reactions were incubated for 1 h at 4 °C. The beads were then incubated in 200 μl elution buffer at 65 °C for 20 min to elute immunoprecipitated materials. We performed duplicate ChIP-seq assays using chromatin from at least two different cell cultures. The ChIP-seq libraries were prepared using NEBNext ChIP-seq Library Prep Master Mix Set for Illumina (NEB catalogue number E6240L) and then run on the Illumina sequencer HS2000. The antibodies used were as follows: Kaiso (Abcam, ab12723), TCF7L2 (Santa Cruz, sc-8631) and Sox10 (Abcam, ab155279). The primers used for ChIP–PCR are listed in Supplementary Table 1.

### ChIP-seq peak-calling and data analysis

All sequencing data were mapped to rat genome assembly rn5 and peak calling was performed as previously described^27^ using MACS (Model-based Analysis of ChIP-seq) version 1.4.2 (http://liulab.dfci.harvard.edu/MACS) with default parameters, to get primary binding regions. To ensure that our data were of high quality and reproducibility, we called peaks with enrichment ≥10-fold over control (*P*≤10^−9^) and compared the peak sets using the ENCODE overlap rules^66^. These identified primary regions were further filtered using the following criteria, to define a more stringent protein–DNA interactome: (1) the *P*-value cutoff was set to <10^−9^; (2) an enrichment of 5-fold and tag number >20, and subtracted with background regions.

The genome-wide distribution of protein binding regions was determined by CisGenome2.0 (http://www.biostat.jhsph.edu/˜hji/cisgenome) in reference to Ensembl RGSC3.4.61 release. This information was also used to group binding regions by the distance between peak summits and TSS. Venn diagrams were constructed using 3Venn Applet. *De novo* motif discovery was performed using Homer software and novel motifs were compared with JASPAR database (http://jaspar.genereg.net). For all ChIP-seq data sets, WIG files were generated with MACS, which were subsequently visualized using Mochiview v1.46. Tcf7l2 ChIP-seq heatmaps were ordered by strength of binding. The heatmaps were drawn using the Heatmap tools provided by Cistrome (http://cistrome.org/ap).

### Gene ontology and enriched motif identification

For ChIP-seq data, binding peaks in rn5 were annotated with MACS 1.4.2. Functional classification of annotated binding genes from ChIP-seq and differentially expressed genes in RNA-seq data was performed using ToppGene (https://toppgene.cchmc.org/). For RNA-seq data, functional classifications were performed using DAVID (http://david.abcc.ncifcrf.gov). Enriched motifs of Tcf7l2-binding peaks in mOLs and iOLs were identified by HOMER (http://homer.salk.edu/homer/ngs/peakMotifs.html). The script ‘findMotifsGenome.pl' was run for ‘Homer Known Motif Enrichment Results' with default parameters.

### Cholesterol supplementation in OPC culture and *in vivo*

Primary mouse OPCs from P5 to P6 cortices of control and *Tcf7l2*ΔHMG pups were prepared by immunopanning with antibodies Ran-2, GalC and PDGFRα sequentially as previously described^65^. Cholesterol (Sigma, C4951) were prepared in ddH2O and added into the culture medium at 10 μg ml^−1^. Cells in growth medium without Platelet-derived growth factor-AA were treated with cholesterol or vehicle control for 4 days and fixed in 4% PFA for 10 min, and stained as described.

For the cholesterol-enriched diet feeding experiment, pregnant female mice were separated into two groups and fed with 2% cholesterol diets (Harlen, TD.01383) or normal diet, respectively, from the time of gestation when plugs were detected after initial mating. The control and *Tcf7l2*ΔHMG pups were kept together with their mothers and fed through lactation until harvested at the indicated time point.

### LPC-induced demyelinating injury in the spinal cord

LPC-induced demyelination was carried out in the ventrolateral spinal white matter of ∼8-week-old mice. Anaesthesia was induced and maintained by peritoneal injection of a mixture of ketamine (90 mg kg^−1^) and xylazine (10 mg kg^−1^). After exposing the spinal vertebrae at the level of T9–T12, meningeal tissue in the intervertebral space was cleared and the dura was pierced with a dental needle. One per cent LPC (L-a-lysophosphatidylcholine; 0.5 μl) via a Hamilton syringe attached to a glass micropipette was injected into the ventrolateral white matter via a stereotactic apparatus. Spinal cord tissues carrying the lesions were collected at time points as follows: 7 dpl, representing peak OPC recruitment, and 14 dpl, representing OL differentiation and new myelin sheath formation (at least 5 mice per control and mutant groups were used for each time point analysis).

### Electron microscopy

Electron microscopy was performed essentially as previously described^22^. Anaesthetized mice were perfused briefly with 0.1 M cacodylate and followed by 2.5% glutaraldehyde/2.5 PFA in 0.1 M cacodylate (pH 7.2). The optic nerves and spinal cord were removed and fixed in fresh fixative overnight at 4 °C. Tissues were rinsed in PBS, postfixed in 1% OsO_4_ in PBS for 1 h, dehydrated in a graded ethanol series, infiltrated with propylene oxide and embedded in Epon. Semi-thin sections were stained with toluidine blue and thin sections were stained with lead citrate.

### Statistical analysis

All analyses were done using Microsoft Excel or GraphPad Prism 6.00 (San Diego, CA, www.graphpad.com). Quantifications were carried from at least three independent experimental groups and data were presented as means±s.e.m. Student's two-tailed *t*-tests and one-way analysis of variance analysis with a Newman–Keuls multiple comparison test were used to compare two data sets and more than two data sets, respectively. *P*-value<0.05 was considered to be statistically significant.

## Additional information

**Accession codes:** All the RNA-seq and ChIP-seq data have been deposited in the NCBI Gene Expression Omnibus (GEO) under accession number GSE65120.

**How to cite this article:** Zhao, C. *et al*. Dual regulatory switch through interactions of Tcf7l2/Tcf4 with stage-specific partners propels oligodendroglial maturation. *Nat. Commun.* 7:10883 doi: 10.1038/ncomms10883 (2016).

## Supplementary Material

Supplementary Information Supplementary Figures 1-12, Supplementary Table 1
