# Supplementary material for: Dual regulatory switch through interactions of Tcf7l2/Tcf4 with stage-specific partners propels oligodendroglial maturation
Source: Nat Commun. 2016 Mar 9;7:10883. doi: 10.1038/ncomms10883 (PMC4786870; doi:10.1038/ncomms10883)
Supplement: Supplementary Information — Supplementary Figures 1-12, Supplementary Table 1 [file ncomms10883-s1.pdf]

SUPPLEMENTARY INFORMATION
Zhao et al.

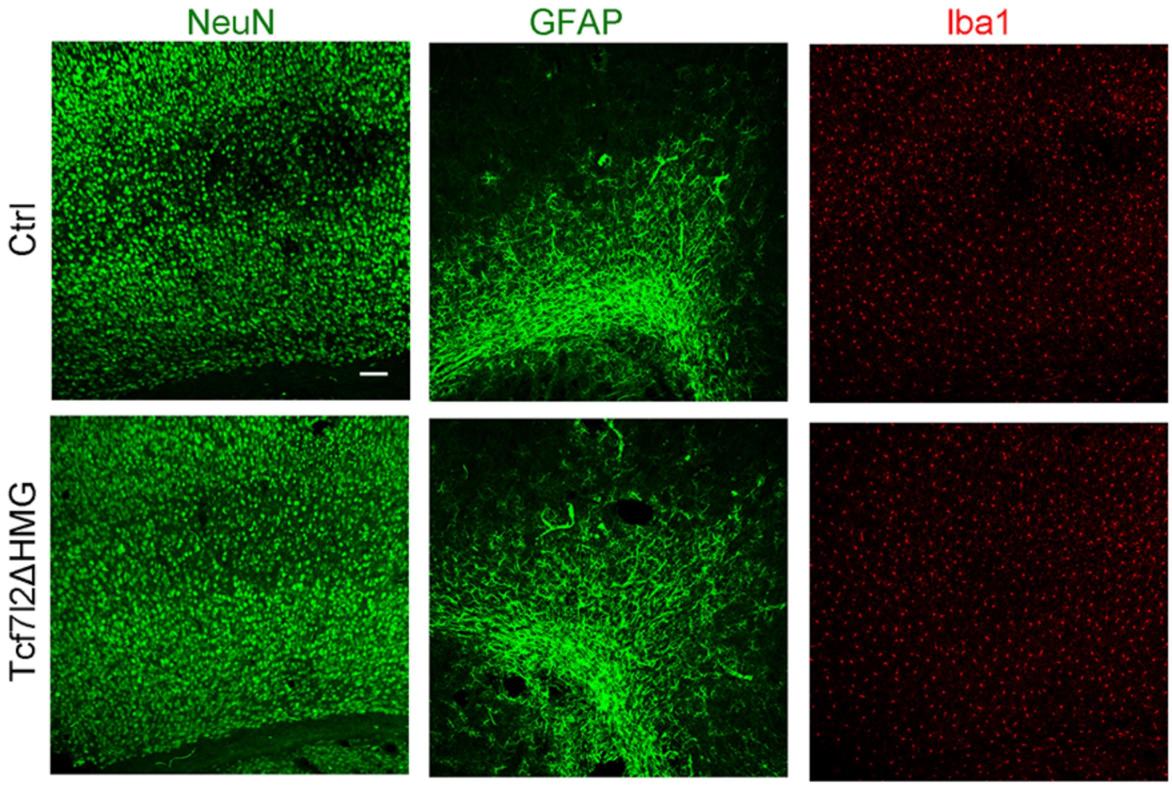

Supplementary Figure 1. ***Tcf7l2*** **ablation in Olig1-Cre-expressing cells does not affect the formation of neurons, astrocytes, or microglia**

The cortices of control (Ctrl) and *Tcf7l2*ΔHMG mice at P14 were immunostained with the pan-neuronal marker NeuN, astrocyte marker GFAP, and microglia marker Iba1 as indicated. Scale bar, 100 µm.

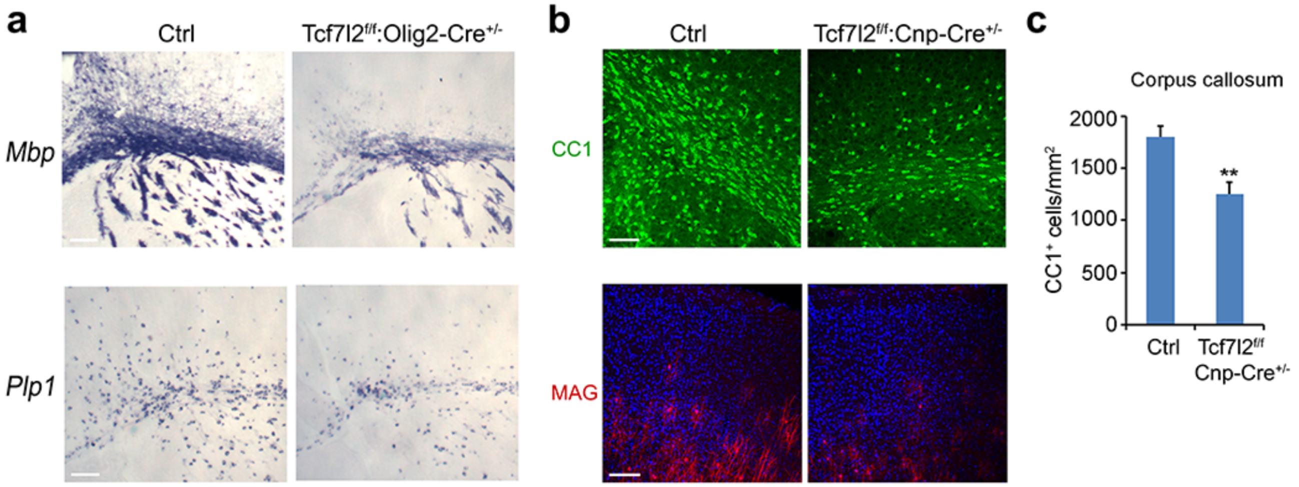

Supplementary Figure 2. ***Tcf7l2*** **ablation by** ***Olig2*****-Cre or** ***CNP*****-Cre-expressing cells impairs OL differentiation.**

(a) *In situ* hybridization analysis of *Mbp* and *Plp1* in cortical sections of control and *Tcf7l2*$^{f/f}$:*Olig2*-Cre mice at P14. Scale bar, 100 µm.

(b) Expression of CC1^{+} and MAG^{+} myelinating cells by immunostaining in the cortex of control and *Tcf7l2*$^{f/f}$:*CNP*-Cre mice at P14. Scale bar, 50 µm.

(c) Quantification of CC1^{+} OLs (per mm^{2}) in the corpus callosum of control and *Tcf7l2*ΔHMG at P14. n = 3 animals/genotype (** $p < 0.01$; Student's *t* test).

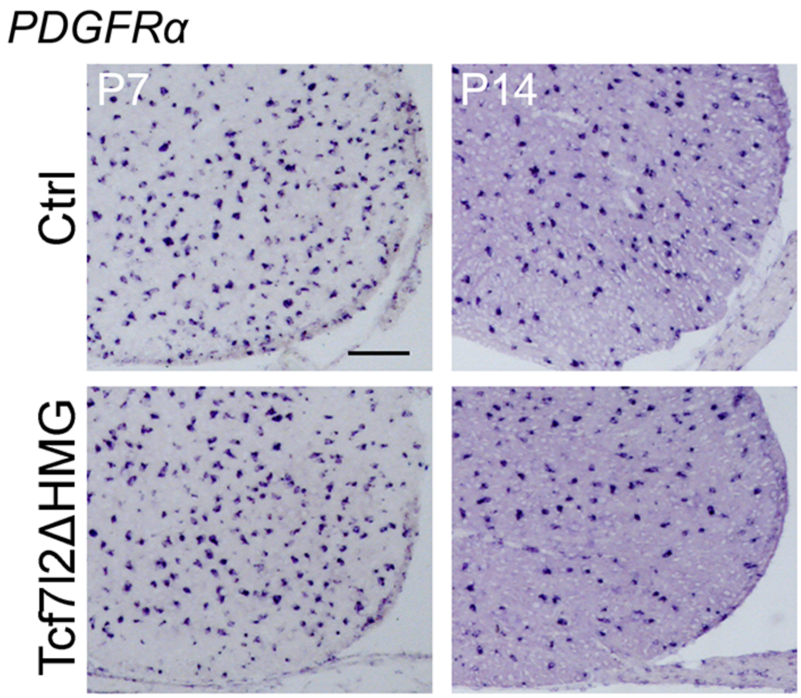

Supplementary Figure 3. ***Tcf7l2*** **ablation in oligodendrocyte lineage cells do not affect OPC development in the spinal cord**

Expression of *PDGFRα* in the spinal cord from control (Ctrl) and *Tcf7l2*ΔHMG mice at P7 and P14 by *in situ* hybridization. Scale bar, 50 µm.

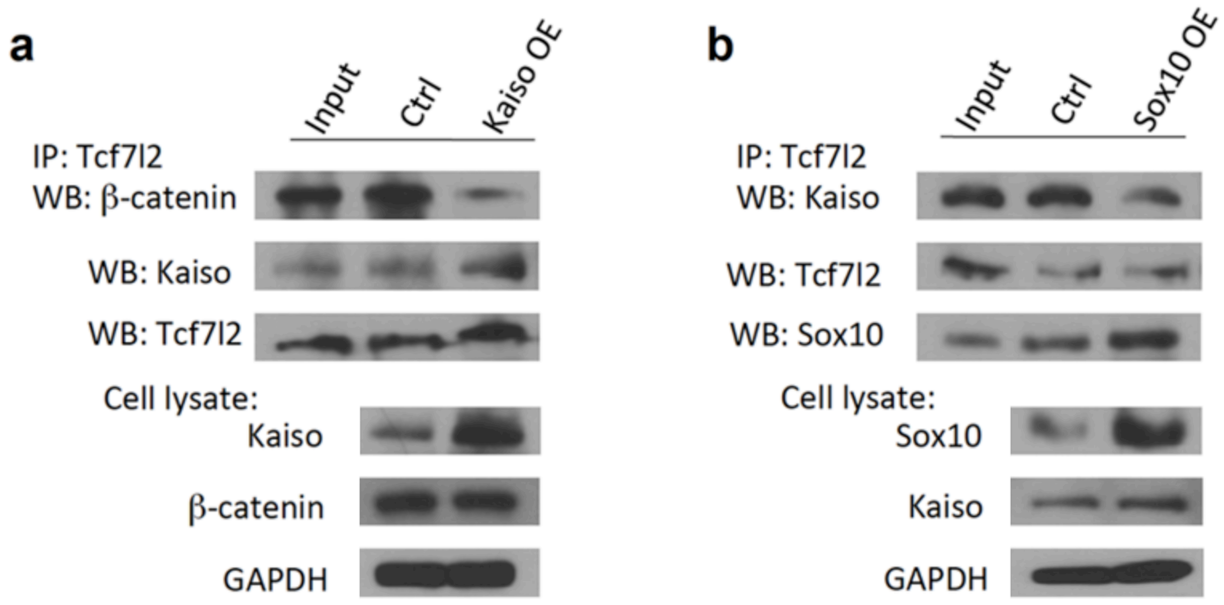

Supplementary Figure 4. Competitive binding among Kaiso, Sox10, β-catenin and Tcf7l2

(**a**) Kaiso competes with β-catenin interaction with Tcf7l2 in Oli-neu cells. The control and pCS2-*Kaiso* were transfected into Oli-neu cells for 48 hr. Lysates were co-immunoprecipitated with anti-Tcf7l2 antibody. Co-immunoprecipitates and cell lysates were subjected to western blot analysis with indicated antibodies. OE, overexpression. GAPDH was used as a loading control.

(**b**) Sox10 competes with Kaiso to interact with Tcf7l2 in Oli-neu cells. The control and pcDNA3-Sox10 were transfected into Oli-neu cells for 48 hr. Cell lysates were co-immunoprecipitated with anti-Tcf7l2 antibody. Co-immunoprecipitates and cell lysates were subjected to western blot analysis with indicated antibodies. GAPDH was used as a loading control.

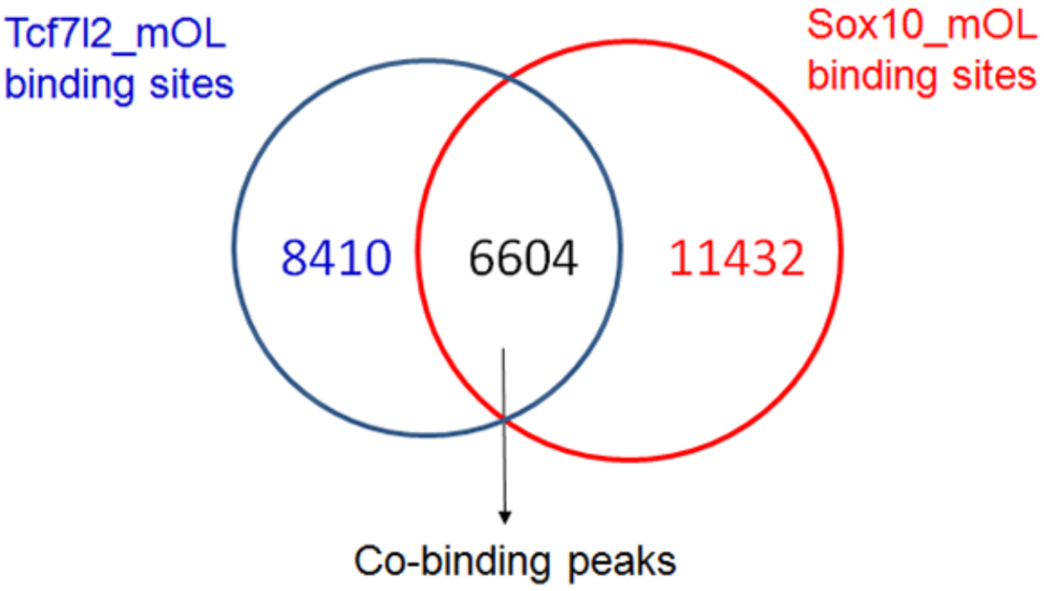

Supplementary Figure 5. Overlapping Tcf7l2 and Sox10 genome occupancy in mOLs
A Venn diagram shows a 44% overlap of Tcf7l2 and Sox10 genome occupancy in mOLs.

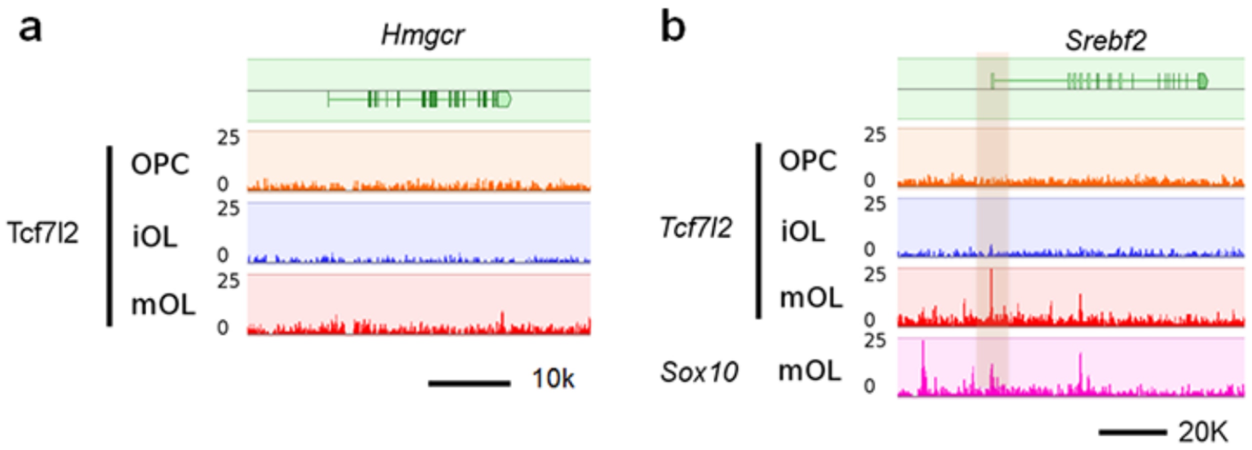

Supplementary Figure 6. Tcf7l2 and Sox10 occupancy on *Hmgcr* and *Srebf2* genes in oligodendrocyte lineage cells

Genome browser visualization of Tcf7l2 and Sox10 binding profiles on the promoter regions of 3-Hydroxy-3-methylglutaryl-CoA reductase (*Hmgcr*) gene (**a**) and Sterol regulatory element binding transcription factor 2 (*Srebf2*) gene (**b**) in OPCs, iOLs and mOLs.

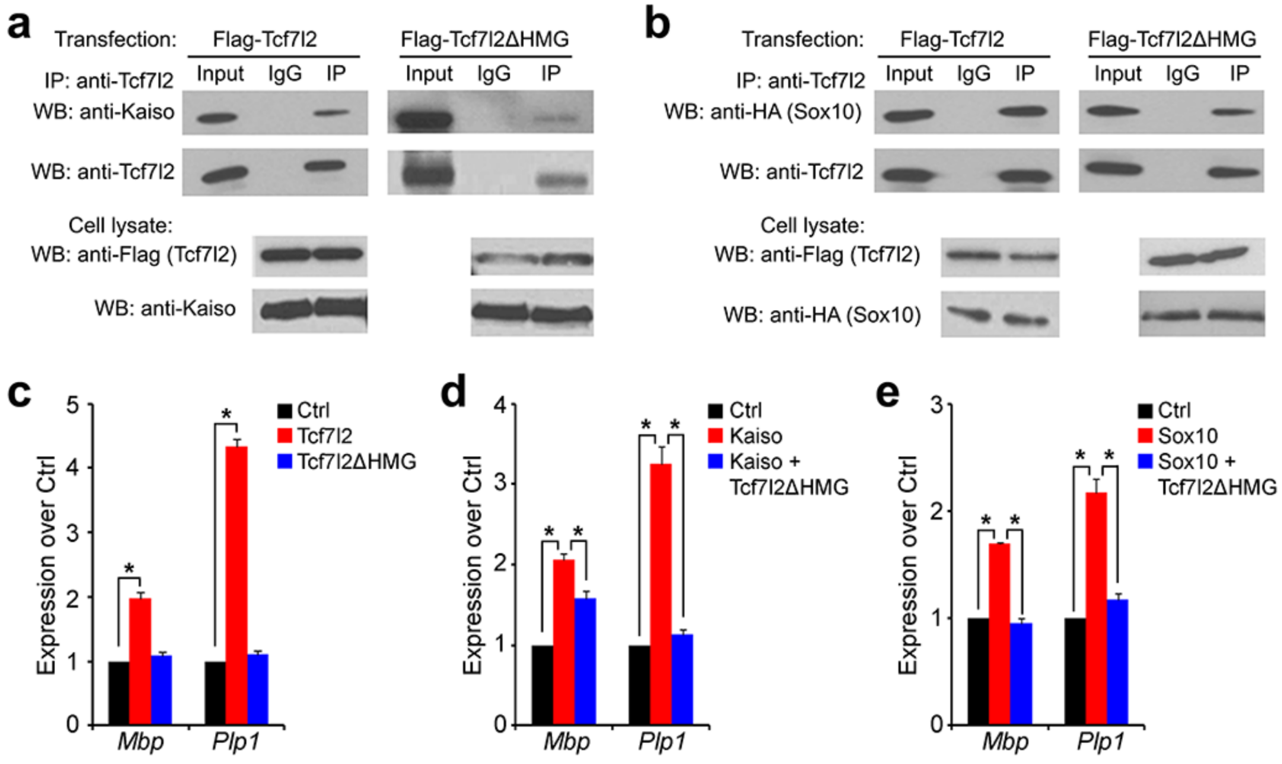

Supplementary Figure 7. Tcf7l2ΔHMG interacts with Kaiso and Sox10 and inhibits their activity for promoting myelin gene expression

(**a,b**) Expression vectors carrying Flag-tagged Tcf7l2 and Tcf7l2ΔHMG were co-transfected with an vector expressing Myc-tag Kaiso (**a**) or HA-tagged Sox10 (**b**) into 293T cells for 48 hr. Lysates were co-immunoprecipitated with anti-Tcf7l2 antibody. Co-immunoprecipitates and cell lysates were subjected to western blot analysis with indicated antibodies. OE, overexpression.

(**c**) qRT-PCR analysis of expression of myelin genes in Oli-neu cells transfected with pcDNA3-control, *Tcf7l2*, and *Tcf7l2*ΔHMG. Data represent the means ± SEMs from three animals/genotype (*$p < 0.05$, **$p < 0.01$; Student's t test).

(**c,d**) qRT-PCR analysis of expression of myelin genes in Oli-neu cells transfected with pCS2-*Kaiso* or *Sox10*, or together with *Tcf7l2*ΔHMG. Data represent the means ± SEMs from three animals/genotype (*$p < 0.05$, **$p < 0.01$; Student's t test).

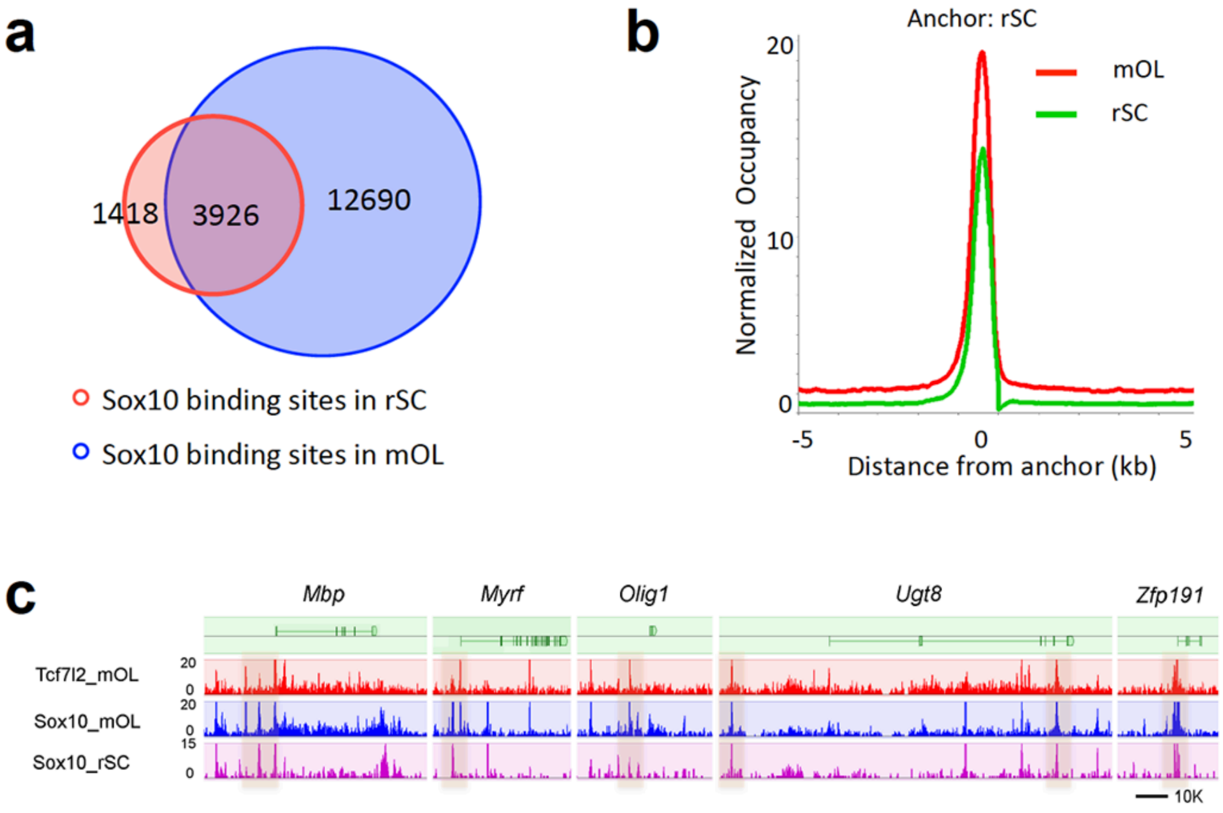

Supplementary Figure 8. Sox10 genomic occupancy in cultured oligodendrocytes and spinal cord

(**a**) The Venn diagram shows that the majority of Sox10 binding sites in spinal cord tissues (SC) overlap with those in cultured mOL. Notably, detected binding sites appear more prolific in isolated OLs than the spinal cord. This could likely be due to differential binding under *in vivo* and *in vitro* conditions or the smaller proportion of OLs in the spinal cord, which may engender a lower signal to noise ratio, leading to an underestimation of binding sites in the intact tissues producing low signal amplitudes.

(**b**) ChIP-seq binding profiles of Sox10 in mOLs around Sox10 peak summits in rat spinal cord.

(**c**) Representative genome browser view of the distribution of Tcf7l2 and Sox10 binding on the promoter regions of myelin genes (*Mbp, Myrf, Cldn11, Ugt8, and Zfp191*) in both mOLs and rat spinal cord (SC) tissues. Representative commonly targeted sites are highlighted.

(d) Genome browser depicts that the binding intensity of Sox10 on the promoter regions of a set of myelination-associated genes (e.g. *Olig1, Plp1, Mag, Gpr37, and Ubl3*) is higher in mOLs than that in rat spinal cord (SC) tissues.

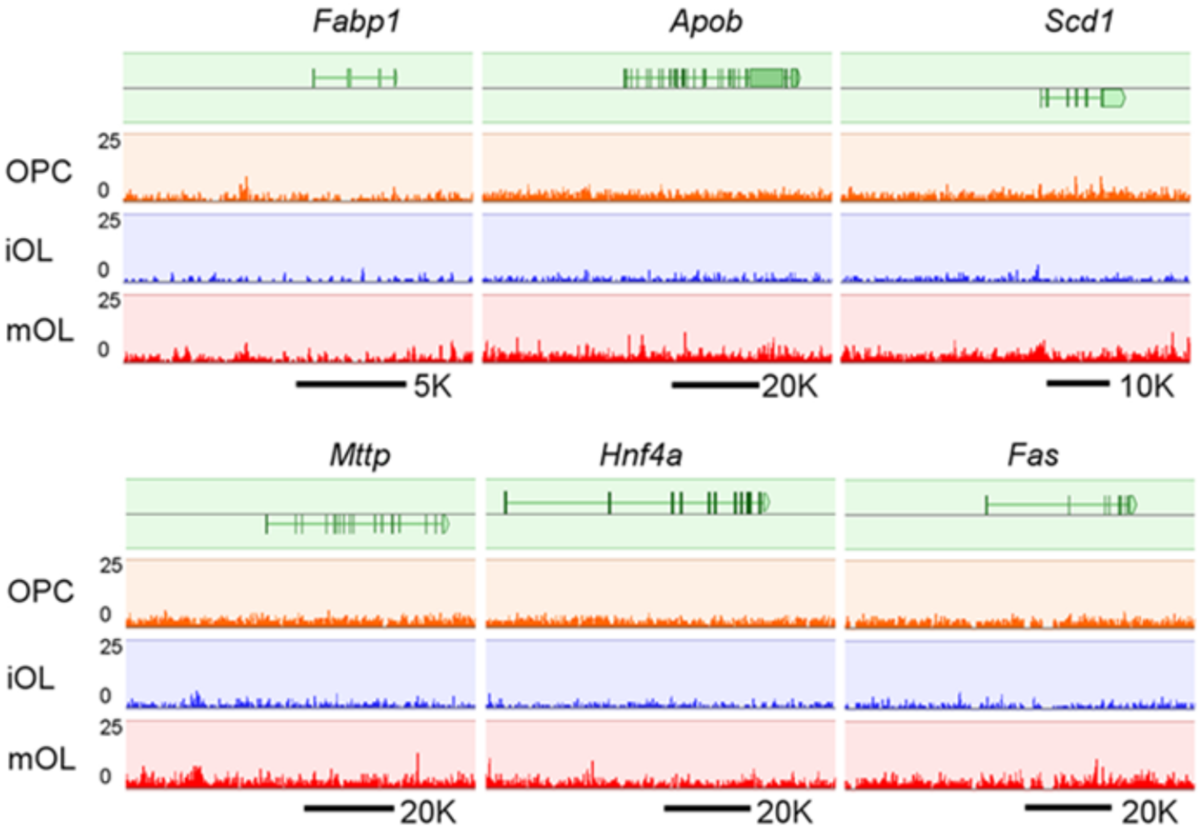

Supplementary Figure 9. Tcf7l2 occupancy on lipid biosynthesis and metabolism genes in oligodendrocyte lineage cells

Genome browser visualization of Tcf7l2 binding profiles in the promoter regions of lipid metabolism genes in OPCs, iOLs and mOLs including *Fabp1*, encoding liver-specific fatty-acid binding protein-1; *Apob*, encoding apolipoprotein B; *Scd1*, encoding stearoyl-CoA desaturase-1; *Mttp*, encoding microsomal triglyceride transfer protein; *Hnf4a*, encoding hepatocyte nuclear factor 4 alpha, and *Fas*, encoding fatty acid synthase.

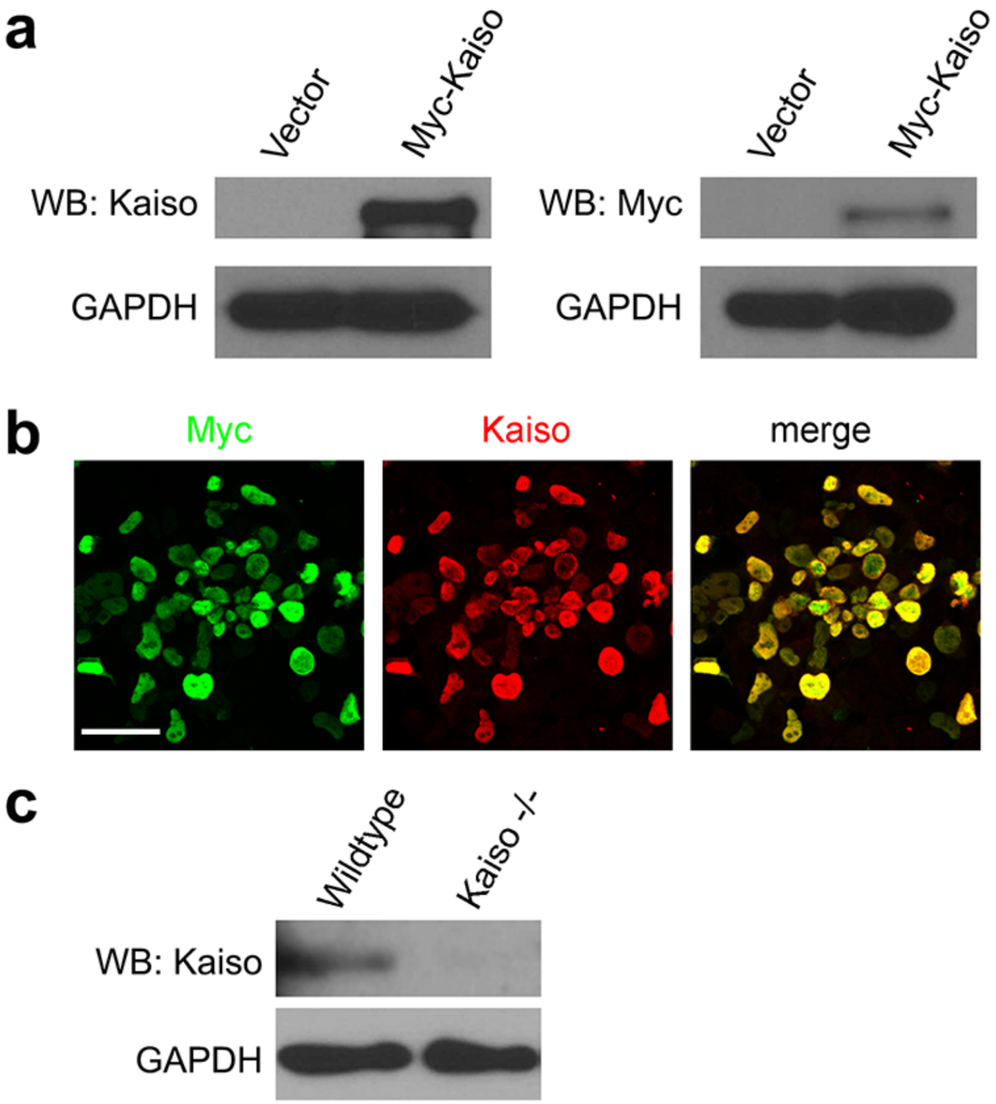

Supplementary Figure 10. Validation of anti-Zbtb33 specificity
(**a**,**b**) 293T cells were transfected with a Myc-tag-Kaiso expression vector and assayed by western blotting (**a**) and immunostaining (**b**) with anti-Kaiso (red) and anti-Myc (green). Scale bar, 50 µm.
(**c**) The adult brain tissues of wildtype and Kaiso null mutants were subject to western blot analysis with anti-Kaiso. GAPDH as a loading control.

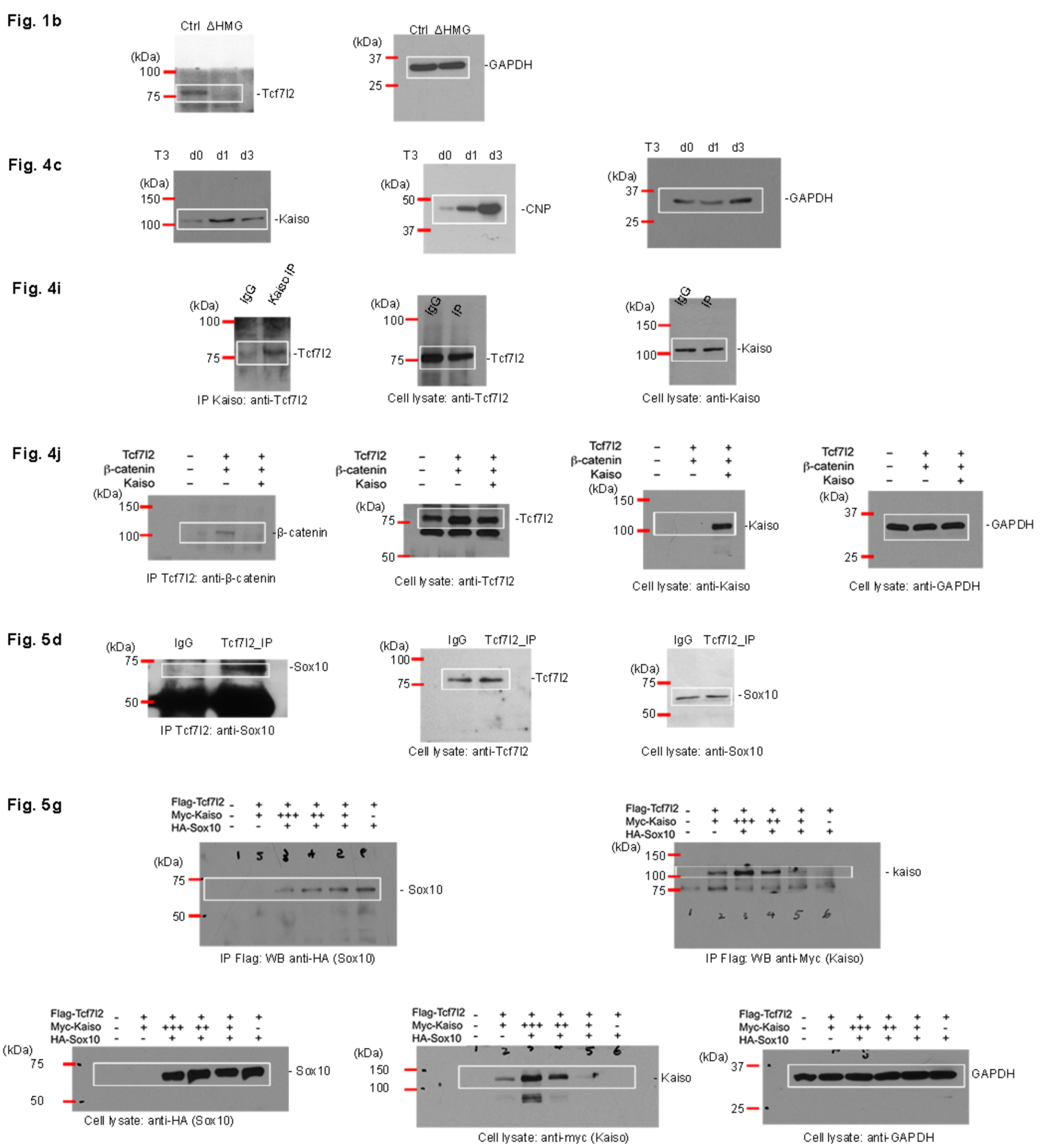

Supplementary Figure 11. Full scans of western blots in main Figures.

Figure 1b, showing wildtype Tcf7l2 and Tcf7l2ΔHMG blot and GAPDH control;
Figure 4c, showing Kaiso, CNP, and GAPDH blots in OPC and OL treated with T3 for 1 and 3 days;
Figure 4i, showing Kaiso immunoprecipitated with Tcf7l2 in cultured iOLs and cell lysate controls;
Figure 4j, showing that Kaiso competes with beta-catenin to interact with Tcf7l2 in 293T cells transfected with indicated expression vectors;
Figure 5d, showing Sox10 immunoprecipitated with Tcf7l2 in cultured mOLs and cell lysate controls;
Figure 5g, showing that Sox10 competes with Kaiso to interact with Tcf7l2 in 293T cells transfected with indicated expression vectors.

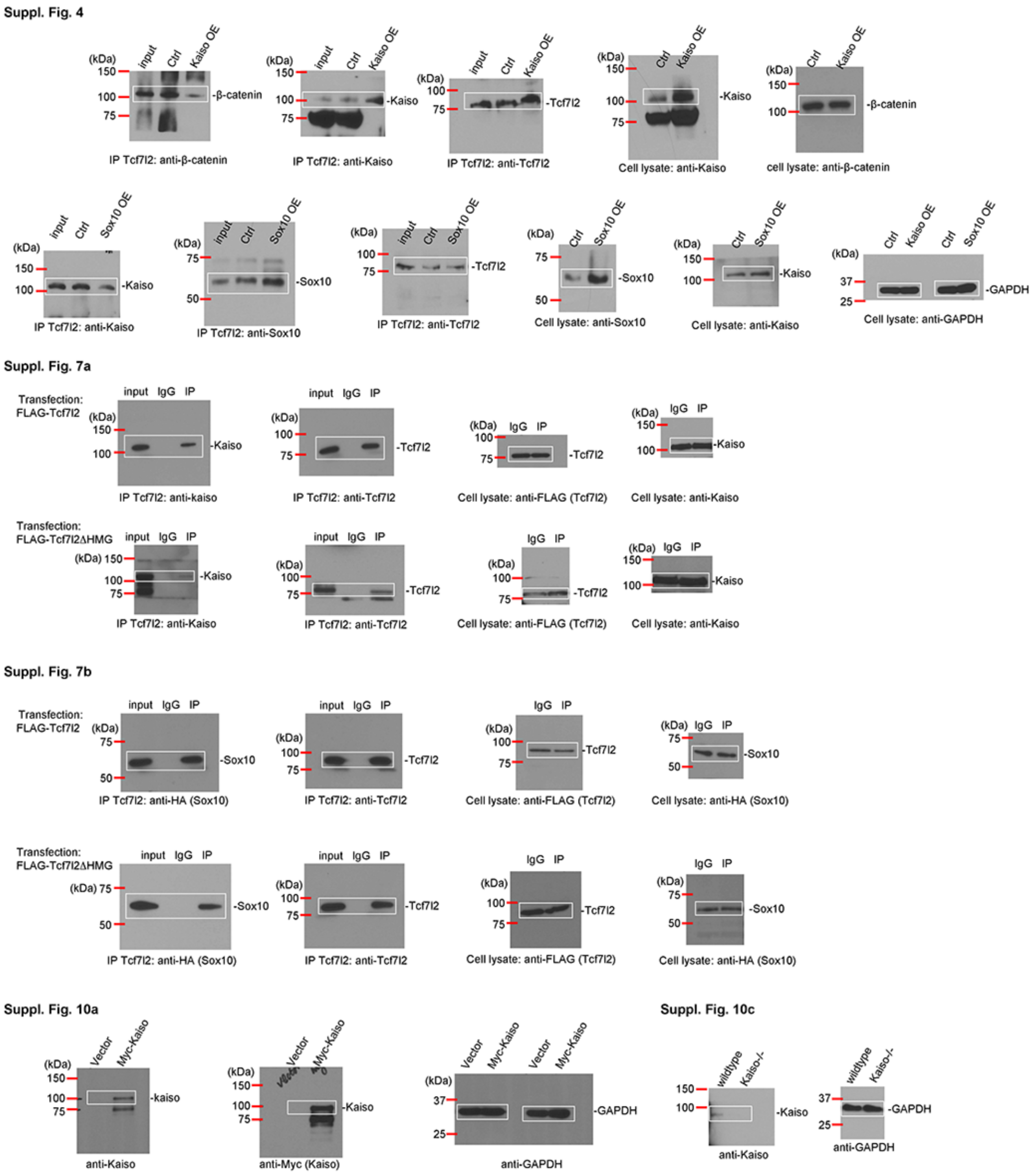

Supplementary Figure 12. Full scans of western blots in Supplementary Figures

Suppl. Fig. 4, showing competitive binding among Kaiso, Sox10, β-catenin and Tcf7l2 in Oli-neu cells transfected with indicated expression vectors;
Suppl. Fig. 7a, showing wildtype Tcf7l2 interaction with Kaiso and Sox10 in transfected 293T cells;
Suppl. Fig. 7b, showing Tcf7l2ΔHMG interaction with Kaiso and Sox10 in transfected 293T cells;
Suppl. Fig. 10a, showing Kaiso antibody recognition of the overexpressed Myc-Kaiso;
Suppl. Fig.10b, showing Kaiso antibody specificity validated with control and Kaiso null mouse brain tissues.

Supplementary Table 1

Primer sequences for qRT-PCR

gene	Species	Forward	Reverse
Gapdh	mouse	tgccaaatatgatgacatcaagaa	ggagtgggtgtcgctgttg
Tcf712 exon11	mouse	tccagggaagaacaggcaaa	tagttatcccgtgcagacca
Sox10	mouse	gttggtacttgtagtccggatg	gtaccctcacctccacaatg
Plp1	mouse	tgctcggctgtacctgtgtacatt	tacattctggcatcagcgcagaga
Cnp	mouse	tccacgagtgcaagacgctattca	tgtaagcatcagcggacaccatct
Mbp	mouse	tcacagaagagaccctcaca	gccgtagtgggtagttcttg
Myrf	mouse	cagacccaggtgctacac	tcctgcttgatcattccgttc
Idh1	mouse	gactcagtagcccaaggttatg	tttctggtacatgcggtagtg
Zbtb20	mouse	ctgtcagtaacagctccgataag	gggtttctgtctggcgtaaata
Bmp7	mouse	ggaattcttccaccctcgatac	ctcccggatgtagtccttataga
Hes1	mouse	tggaatgccgggagctatctttct	tggaatgccgggagctatctttct
Kaiso	mouse	gttccaaagttgtccgtgttag	ggacaccaagctctgctataa
Tcf7l2	mouse	atggttagtaccacagcaagg	gggagggaacctagacataga
Id2	mouse	atggaaatcctgcagcacgtc	tggttctgtccaggtctct
Fdps	mouse	tcgggtgaaagcactgtatg	gcactgctctatgagactcttg
Fdft1	mouse	cccatagttggtgaagacatagag	cttcctgttggtcttccagataa
Lss	mouse	ggaaggactcaacaccctattc	cagtaactcatgggcagatagac
Cyp51	mouse	aggatctgcctcccttaact	ggcctcagtcttagtgtttctt
Hsd17b7	mouse	tcaaccagaagggtctgtattc	gtaggagcaacgtccagataaa
Dhcr24	mouse	gagtcatcgtcccacaagtatg	ggcatagaacaggtctgagttt
Srebf2	mouse	tggatgacgcaaaggtcaa	caggaaggtgaggacacataag
Hmgcr	mouse	ctcatgaacgtggtgtgtctat	gctcccatcaccaaggaataa
Gapdh	rat	tccagtatgactctacccacg	cacgacatactcagcaccag
Mbp	rat	ttgactccatcgggcgcttcttta	ttcatcttgggtcctctgcgactt
Cnp	rat	ctactttggcaagagacctcc	agagatggacagtttgaaggc
Plp1	rat	tctttggcgactacaagaccacca	caaacaatgacacacccgctccaa
Mag	rat	acagcgtcctggacatcatcaaca	atgcagctgacctctacttccgtt
Id2	rat	atggaaatcctgcagcacgtcatc	acgtttggttctgtccaggtctct
Tcf7l2	rat	atggttagtaccacagcaagg	gggagggaacctagacataga
Kaiso	rat	ctgctgaactccctgaatgaa	ctgagaagagctgatggaagtaag
Hes5	rat	accagcccaactccaaac	agtaaccctcgctgtagtcc
Myrf	rat	actgccaacaacatgcggaagaag	tgggttagaggcccgaacaatgat
Id4	rat	actgtgcctgcagtgcgatatgaa	tgc agg atc tcc act ttg ctg act
Sp5	rat	ttcgtgtgcaactggctctt	aggtgatcgcttcgcatgaa
Wnt10a	rat	actccgacctggtctacttt	gacccgtgctgctcttattg
Wnt4	rat	gaagaggaaacgtgcgagaa	tggtattggcactcctcaatg
Wnt11	rat	aacaggatcccaagccaataa	catggcacttacacttcgtttc
Dhcr24	rat	cgtggaagggttgctgtatt	gaagaaccacggcttgtagtaa
Lss	rat	acactgggctggtgattatg	ccgtaccatttcctctctgtatc

Cyp51	rat	aggatctgcctcccttaact	ggcctcagtcttagtgtttctt
Hsd17b7	rat	ctctcaaccctctgaccaaatac	gtgtcttcatctacgtccatctt
Axin2	rat	caacgacagcgagttatcca	ctctctctggagctgtttcttac
Hes1	rat	agaaaaattcctcgtccccg	tttcatttattcttgcccggc

Primer sequences for ChIP-qPCR

Rat gene	Forward	Reverse
Ctnnb1	cagacagcaagctgaagaga	ggcttcctaaatcccagacaa
Ccnd1	ctggaggctgcaggacttt	ttctctgcccggctttgat
Wnt11	ctgacgtgggcggatcaaa	atctccggagctctctcct
Wnt10a	gtagcagctgactccacttag	gggcttgttaagatgcacaattag
Lef1	gactggctgggatccatattc	ctgcggctttcatcctttct
Axin2	gaggtctccgcaaggagtg	ggcctgccaacttcaaagc
Sp5	gagccgctattctttgatgattg	agaatttgataagggctttgctg
Control	ttacaggcacataggaggtaaa	gaatgcatacaccaccacaag
Myrf	gctccagtgtctgtgctatg	cccagcatcctgttccattta
Fdps	caagatgtttgcggcttacac	aggctgggagtcgtagtatt
Fdft1	gattggagggctggaaagaa	tggtctctacacctacacctc
Lss	gccaatcagagcatcacca	taggcccagcctccaaa
Cyp51	attcttggagccctcactttc	cgcctccagctaggtact
Hsd17b7	ggagtctttgttctgagtgagg	agccacctttgacttggatt
Dhcr24	tctagtctgggctactccattt	atgaagaatgtgtgtgggagtt
Srebf2	ctggaactgatccaacggaaa	ctcagatttgcatagcgagact

Primer sequences for promoter cloning

Rat gene	Forward	Reverse
Cyp51 promoter	cgctgaatagctgcagaggc	tcctgtcacctgctccatcg
Hsd17b7 promoter	tgtcaccgagcacccagtgag	tacagcagcctgataggcac
Lss promoter	agcgagaggaagcagccttc	caaccctggaggcttcccat
Dhcr24 promoter	ctaacccaagataatataatc	aggaagaggcacacaaaca
Hmgcs1 promoter	gctgctctggaaactagaaag	aaacccgctccctgagccctat
Hmgcr promoter	agcgcgagacccgctgcc	gcacccgagagtgggaag
Fdft1 promoter	tgagagaccgaacaagctc	gggattgcaggtgtgagctt
Fdps promoter	ccgtaactccgacacgactg	ccttgcgcttcctaggcaag
Srebf2 promoter	gtctcggatgggacaagtgtg	ttctcatccatcgcccagttc

Primer sequences for generating *Tcf7l2*ΔHMG via mutagenesis

cctgcgcccgagaatctggt
gggaagaagaagaagagaaaaagagacaagcag
